# Biochar–Urea Peroxide Composite Particles Alleviate Phenolic Acid Stress in *Pogostemon cablin* Through Soil Microenvironment Modification

**DOI:** 10.3390/microorganisms13122772

**Published:** 2025-12-05

**Authors:** Yuting Tu, Baozhu Chen, Qiufang Wei, Yanggui Xu, Yiping Peng, Zhuxian Li, Jianyi Liang, Lifang Zhuo, Wenliang Zhong, Jichuan Huang

**Affiliations:** 1 Institute of Agricultural Resources and Environment, Guangdong Academy of Agricultural Sciences, Guangzhou 510640, China; 2 Key Laboratory of Plant Nutrition and Fertilizer in South Region, Ministry of Agriculture and Rural Affairs of the People’s Republic of China, Guangzhou 510640, China; 3 Guangdong Key Laboratory of Nutrient Cycling and Farmland Conservation, Guangzhou 510640, China; 4 School of Chemistry and Environment, Guangdong Ocean University, Zhanjiang 524088, China; 5 The College of Natural Resources and Environment, South China Agricultural University, Guangzhou 510642, China

**Keywords:** *Pogostemon cablin*, continuous cropping, phenolic acids, biochar, urea peroxide, soil microbial community

## Abstract

The continuous-cropping obstacles of *Pogostemon cablin* (patchouli) is severely constrained by autotoxic phenolic acids accumulated in the rhizosphere soil. Biochar adsorption and chemical oxidation are common remediation strategies; they often fail to simultaneously and efficiently remove phenolic allelochemicals while improving the soil micro-ecological environment. To address this issue, this study developed a novel biochar–urea peroxide composite particle (BC-UP). Batch degradation experiments and electron paramagnetic resonance (EPR) analysis confirmed the synergistic adsorption-oxidation function of BC-UP. A pot experiment demonstrated that application of BC-UP (5.0 g/kg) significantly alleviated phenolic acid stress. Specifically, BC-UP application significantly enhanced shoot biomass by 28.8% and root surface area by 49.3% compared to the phenolic acid-stressed treatment and concurrently reduced the total phenolic acid content in the rhizosphere soil by 37.3%. This growth promotion was accompanied by the enhanced accumulation of key bioactive compounds (volatile oils, pogostone, and patchouli alcohol). BC-UP amendment also improved key soil physicochemical properties (e.g., pH, and organic matter) and enhanced the activities of critical enzymes. Furthermore, BC-UP reshaped the microbial community, notably reducing the fungi-to-bacteria OTU ratio by 49.7% and enriching the relative abundance of Firmicutes and Nitrospirota but suppressing the Ascomycota phylum abundance. Redundancy analysis identified soil sucrase and catalase activity, total phenolic acid content, and Ascomycota abundance as key factors influencing patchouli biomass. In conclusion, BC-UP effectively mitigates phenolic acid stress through combined adsorption and radical oxidation, subsequently improving soil properties and restructuring the rhizosphere microbiome, offering a promising soil remediation strategy for patchouli and other medicinal crops.

## 1. Introduction

Continuous cropping obstacles pose a significant threat to global agriculture, undermining soil health and crop sustainability. Patchouli (*Pogostemon cablin*), a valuable medicinal plant rich in bioactive compounds like patchouli alcohol, is extensively used in traditional Chinese medicine, cosmetics, and even in the prevention of COVID-19 [1,2,3]. However, driven by high market demand and limited arable land, patchouli cultivation faces a severe challenge: it is highly sensitive to continuous cropping. This practice leads to stunted growth, elevated disease incidence, and a high mortality rate (>30%) in replanted cuttings [4]. To compensate, growers often increase chemical inputs, which exacerbates soil degradation and compromises herb quality, creating a vicious cycle [5]. This phenomenon is not unique to patchouli; it affects approximately 70% of rhizome-based medicinal herbs, such as *Panax ginseng*, *Codonopsis pilosula*, *American ginseng* (*Panax quinguefolium*), and *Panax notoginseng*, representing a major bottleneck for the herbal medicine industry [6,7,8,9].

Continuous cropping obstacles in medicinal plants arise from the interaction of biotic and abiotic factors, primarily involving allelopathic autotoxicity, deterioration of soil physicochemical properties, and disruption of the soil biological environment [10,11,12]. The accumulation of allelopathic substances, particularly phenolic acids, is a major driver of this phenomenon [13,14]. Wu et al. [15] and Xu et al. [16] isolated and identified 30 compounds from the rhizosphere soil of patchouli and 55 compounds from various parts of the plant. Among them, five allelochemicals, including benzoic acid, vanillic acid, salicylic acid, *p*-hydroxybenzoic acid, and malonic acid, were ultimately identified as key contributors to the continuous cropping obstacles of patchouli. The accumulation of these compounds induces oxidative stress in plants by triggering reactive oxygen species (ROS) bursts, which disrupts cellular signaling, impairs physiological metabolism, and leads to microbial community dysbiosis [5,17,18]. Specifically, phenolic acids can elevate levels of ROS, causing lipid peroxidation, cell membrane damage, and programmed cell death, thereby inhibiting growth [19]. Consequently, developing targeted technologies to mitigate these phenolic acids in soil is crucial for fundamentally alleviating continuous cropping obstacles in patchouli.

To mitigate phenolic acid stress in soil, various soil amendments have been developed in recent years. The efficacy of soil amendments in mitigating continuous cropping obstacles is largely mediated by their interaction with the rhizosphere microbial community. Microorganisms play a dual role: they are not only affected by allelochemical stress but are also pivotal agents in its mitigation. Specific microbial taxa, such as *Pigmentiphaga* and *Acinetobacter*, can directly detoxify allelochemicals through specialized metabolic pathways [20,21]. Furthermore, amendments that improve soil physicochemical properties can indirectly contribute by reshaping the microbial community. This restructuring often enriches beneficial degraders or plant growth promoting rhizobacteria, thereby enhancing overall soil resilience and function [22,23]. Therefore, a promising strategy involves synergizing the physicochemical effects of soil conditioners with the ecological regulation of the rhizosphere microbiome to sustainably overcome phenolic acid stress.

In recent years, biochar and peroxides have emerged as promising soil amendments for mitigating continuous cropping obstacles. Biochar’s porous structure and high adsorption capacity enable it to sequester phenolic acid allelochemicals [24,25]. Its efficacy in regulating microbial communities and enhancing medicinal plant yield is well-established. For instance, biochar application remediated the rhizosphere microbiome in *Radix pseudostellariae* monoculture soil by suppressing the pathogen *Fusarium oxysporum* [26]. Similarly, in a ten-year continuous cropping system for *Panax notoginseng*, biochar at 15 t/ha dramatically increased the plant survival rate from 6.0% to 69.5%. This application also ameliorated soil properties, increasing pH and nutrient availability while reducing concentrations of allelochemicals like vanillic acid [27]. While biochar effectively adsorbs phenolic acids, this process merely transfers them without degradation, entailing risks of secondary release [28]. In contrast, peroxides like urea peroxide offer a destructive approach. Upon decomposition in soil, they generate H_2_O_2_ for Fenton-like reactions with indigenous iron, producing highly oxidative free radicals (e.g., •OH) that efficiently degrade organic pollutants [29,30]. Urea peroxide additionally provides the additional nitrogen supplementation [31,32]. Hence, integrating the adsorptive capability of biochar with the oxidative power of urea peroxide is a promising strategy for the complete removal of allelochemicals; however, such a synergistic system remains underdeveloped.

Therefore, this study developed novel composite particles by coupling biochar with urea peroxide (BC-UP), hypothesizing that this design would enable simultaneous adsorption and oxidation of phenolic acids. The hypothesis was tested through batch degradation and pot experiments with *Pogostemon cablin* (patchouli). The effects of BC-UP on phenolic acid removal, plant growth, soil properties, and the rhizosphere microbial community were systematically evaluated against the phenolic acid stress and individual components. This work aims to provide a novel design strategy for soil amendments to alleviate continuous cropping obstacles in medicinal plants.

## 2. Materials and Methods

### 2.1. Materials

The *Pogostemon cablin* cultivar used in this study was the “Zhanjiang” variety, one of the predominant cultivated varieties in the Guangdong region of China. The phenolic acids used in this research, including *p*-coumaric acid, phthalic acid, salicylic acid, vanillic acid, 4-hydroxybenzoic acid, and benzoic acid, were all of analytical grade and purchased from Aladdin Reagent Co., Ltd. (Shanghai, China). The chromatographic-grade patchouli alcohol and pogostone were obtained from Shanghai Macklin Biochemical Co., Ltd. and Acmec Biochemical Technology Co., Ltd. (Shanghai, China), respectively. Analytical-grade acetone were sourced from Guangzhou Chemical Reagent Factory (Guangzhou, China).

The soil used in the pot experiment was collected from a rice paddy field and is classified as lateritic red soil. The soil pH was 6.41, with an organic matter content of 2.51%. The content of alkali-hydrolyzable nitrogen (AN), available phosphorus (AP), and available potassium (AK) were 92.8, 140.1, and 71.6 mg/kg, respectively. The biochar used in this study was coconut shell-derived activated carbon, also purchased from Guangzhou Chemical Reagent Factory, with a specific surface area of 331.4 m^2^/g, an ash content of 5.6%, and a pH of 7.47 at the point of zero charge. Urea peroxide was synthesized through a crystallization method using hydrogen peroxide and urea as raw materials [33]. Briefly, urea and 30% hydrogen peroxide solution were mixed at a molar ratio (H_2_O_2_:urea) of 1.1:1. The reaction was carried out at 35 °C for 40 min under continuous stirring. Subsequently, the product was crystallized at 2 °C, filtered, and dried. The resultant urea peroxide crystals had a purity of 88.84% as determined by iodometric titration.

The biochar-urea peroxide composite particles (BC-UP) were prepared using a mixed granulation method. The optimal mass ratio of biochar, urea peroxide, and binder was determined to be 4:1:0.26 through a series of single-factor and orthogonal experiments that evaluated the comprehensive performance of the composite granules in terms of compressive strength, water resistance, and phenolic acid removal efficiency. Briefly, powdered biochar, synthesized urea peroxide (purity: 88.84%), and an attapulgite binder were weighed and thoroughly mixed at a mass ratio of 4:1:0.26. Deionized water was then introduced dropwise (accounting for approximately 10–15% of the total solid mass) under continuous mixing until a homogeneous, plastic mixture was achieved. This mixture was then manually shaped into spherical particles with a uniform diameter of 5.0 ± 0.5 mm. The resulting wet BC-UP particles were rapidly dried in an oven at 40 °C for 12 h. The prepared BC-UP composite particles had a water content of 5.6%. Based on the initial mass ratio, the final chemical composition of the BC-UP particles was approximately 71.8% biochar, 17.9% urea peroxide, and 4.7% attapulgite binder. For comparison, control particles consisting solely of biochar (BC) and urea peroxide (UP) were also produced following the same protocol, maintaining the same mass ratio of biochar or urea peroxide to binder mass ratio of 4:0.26. The morphology and surface functional groups of the BC-UP composite were characterized, with the SEM image and FTIR spectrum provided in Figure S1.

### 2.2. Batch Experiment for Phenolic Acid Degradation and Analytical Characterization Methods

To evaluate the degradation capability of BC-UP composite particles, *p*-coumaric acid (*p*-CA) was selected as a representative phenolic acid. The batch experiments were conducted in 250 mL glass conical beakers containing 100 mL of *p*-CA solution (initial concentration: 100 mg/L). Different reaction systems were established by adding specific amounts of particles: 10.0 g/L of BC-UP composite, 8.0 g/L of BC alone, or 2.0 g/L of UP alone, with 0.2 mmol/L Fe^2+^ (in the form of FeSO_4_·7H_2_O) or 2 g/L of lateritic red soil was introduced into the respective systems. All reactions were carried out in a thermostatic shaker bath at 25 °C with a consistent stirring speed of approximately 200 rpm. After 180 min of reaction, the solution was withdrawn and immediately filtered through a 0.22 μm nylon syringe filter prior to HPLC analysis.

The concentration of *p*-CA in the filtrate was quantified using a Waters Alliance e2695 HPLC system equipped with a Waters 2489 UV/visible detector and a Symmetry C18 column (5 μm, 4.6 × 250 mm, Waters Corporation, Milford, MA, USA). The column temperature was maintained at 30 °C. An isocratic elution was performed with a mobile phase consisting of methanol and 0.1% formic acid aqueous solution (30:70, *v*/*v*) at a flow rate of 0.6 mL/min. The injection volume was 10 μL, and the detection wavelength was set at 288 nm. Quantification was achieved using an external standard method.

Electron paramagnetic resonance (EPR) spectroscopy was employed to identify the generated radical species, including the hydroxyl radical (•OH), superoxide radical (•O_2_^−^), and singlet oxygen (^1^O_2_). Measurements were performed on an A300-10-12 spectrometer (Bruker BioSpin GmbH, Rheinstetten, Germany) using 0.1 mol/L DMPO and TEMP as spin-trapping agents.

### 2.3. Pot Experiment Method

The pot experiment with *Pogostemon cablin* was conducted from 17 May to 17 June 2024, in a greenhouse at the Institute of Agricultural Resources and Environment, Guangdong Academy of Agricultural Sciences. Five treatments were established, including (1) conventional cultivation without amendments (CK), (2) phenolic acid stress (PhA), (3) phenolic acid + biochar-urea peroxide composite particles (PhA+BC-UP), (4) phenolic acid + biochar particles (PhA+BC), and (5) phenolic acid + urea peroxide particles (PhA+UP).

In the preliminary study, the phenolic acids in the rhizosphere soil of patchouli were extracted using the method described by Cheng et al. [34]. And a total of 17 phenolic acids were identified through HPLC-MS, including *p*-coumaric acid, phthalic acid, vanillic acid, salicylic acid, 4-hydroxybenzoic acid, benzoic acid, syringic acid, vanillin, 3,4-dihydroxybenzoic acid, caffeic acid, syringaldehyde, trans-cinnamic acid, 3,4-dihydroxybenzaldehyde, trans-ferulic acid, 3-phenylpropionic acid, sinapic acid, and phenylalanine. Among these, the first six phenolic acids accounted for 80.3–82.7% of the total detected phenolic acids, and their content ratios in the rhizosphere soil of patchouli approximately 3.3:3.0:1.5:1.0:1.0:0.7.

Preliminary studies also indicated that when the exogenous phenolic acid application exceeded 100 μg/g would impose significant allelopathic stress on patchouli seedlings. Therefore, in this study, the exogenous phenolic acid application rate was set to 100 μg per gram of soil., with specific amounts of *p*-coumaric acid, phthalic acid, vanillic acid, salicylic acid, 4-hydroxybenzoic acid, and benzoic acid being 31.4, 28.6, 14.3, 9.5, 9.5, and 6.7 μg/g, respectively.

A preliminary dose–response experiment was performed using BC-UP application rates of 2.5, 5.0, 10.0, and 15.0 g/kg (data shown in Table S1). The results indicated that a dosage of 5.0 g/kg was optimal for alleviating PhA-induced stress, as it most effectively restored plant growth metrics, including shoot fresh weight, plant height, and root length, to levels closest to the non-stressed control (CK). Higher doses, particularly 15.0 g/kg, were found to be inhibitory. Therefore, the 5.0 g/kg dosage was identified as the optimal rate and was consequently selected for all subsequent experiments in this study. This optimal dosage (5.0 g/kg) was applied in the PhA+BC-UP treatment. For the PhA+BC and PhA+UP treatments, the application rates of biochar and urea peroxide particles were 4.0 g/kg and 1.0 g/kg, respectively. To ensure consistent nitrogen fertilizer application across all treatments, urea was supplemented in all treatments except for PhA+BC-UP and PhA+UP.

The exogenous additives and mixed phenolic acid solution were thoroughly mixed with 500 g of air-dried soil (sieved to ≤2 mm) and then transferred into square plastic pots (9 cm × 9 cm × 9 cm). Each pot was planted with one rooted 8-week-old patchouli cutting. All cuttings displayed uniform growth characteristics, averaging 10 cm in height with six true leaves and no observable pest or disease symptoms. The experiment followed a randomized complete block design with seven replicates per treatment (one plant per replicate), totaling 35 pots. To minimize the effects of potential environmental heterogeneity within the greenhouse, all pots were initially placed in a completely randomized design on the same bench. and their positions were re-randomized weekly. The plants were grown under natural sunlight without supplemental lighting. Throughout the study, the greenhouse environmental conditions were monitored daily: the air temperature fluctuated between 22 °C and 33 °C, and the relative humidity ranged from 87% to 98%. No artificial climate control was applied beyond standard ventilation. All pots received uniform watering management.

### 2.4. Determination of Physiological and Biochemical Indicators

#### 2.4.1. Determination of Patchouli Growth Indexes

On the 30th day after transplantation, three uniform patchouli seedlings were randomly selected from each treatment group. The fresh weight of the shoot and root parts were measured separately using an analytical balance (Pioneer^TM^ CP4102, OHAUS, Parsippany, NJ, USA) with an accuracy of 0.01 g. Plant height was measured using a stainless steel ruler (graduation: 0.1 mm). Root surface area was analyzed using an Epson Expression 12000XL root scanner (Seiko EPSON Corporation, Nagano, Japan) in combination with WinRHIZO Pro image analysis software (version 2021, Regent Instruments Inc., Quebec City, QC, Canada).

#### 2.4.2. Determination of Patchouli Quality Indexes

Approximately 3 g of chopped patchouli cutting was soaked in 50 mL acetone for 30 min, followed by 2 h ultrasonic extraction at 50–53 °C. The filtrate was concentrated by rotary evaporation, and the residue was dissolved in ether. After transferring to a pre-weighed vial and evaporating the ether, the volatile oil yield was calculated. The extract was redissolved in acetone to 5 mL and stored at 4 °C. Then, patchouli alcohol and pogostone were analyzed by an Agilent 8890-5977B GC-MS system (Agilent Technologies, Santa Clara, CA, USA) following the method of Xu et al. [35]. Compounds were separated on an HP-5 MS UI capillary column (30 m × 0.25 mm × 0.25 μm) with the following temperature program: 60 °C (1 min), 20 °C/min to 110 °C (1 min), 2 °C/min to 120 °C (0.5 min), and 20 °C/min to 250 °C (3.5 min). Helium carrier gas flowed at 1.0 mL/min with an injection volume of 1 μL. The split ratio was 50:1, and the injector temperature was 280 °C.

#### 2.4.3. Determination of Soil Physiochemical Properties and Enzyme Activities

Rhizosphere soil samples from patchouli cuttings were collected using a brush. One portion was air-dried and sieved through a 1 mm mesh for physicochemical analysis, and the other portion was stored at −80 °C for phenolic acid and microbiological determinations. All soil enzyme activities were immediately determined using fresh soil, and the results were uniformly normalized and expressed per gram of dry soil weight based on the measured soil moisture content.

Soil pH was measured potentiometrically at a 2.5:1 water-to-soil ratio. Organic matter content was determined by the potassium dichromate digestion method, alkali-hydrolyzable nitrogen (AN) by the alkaline diffusion method, available phosphorus (AP) by the sodium bicarbonate extraction-molybdenum antimony colorimetric method, and available potassium (AK) by NaHCO_3_ extraction and flame photometry. Soil catalase activity (S-CAT) was determined by quantifying the decomposition of 0.3% H_2_O_2_ via potassium permanganate titration after a 24 h reaction period. Urease activity (S-UE) was assessed through the quantification of ammonia nitrogen released during urea hydrolysis. The NH_3_–N concentration was measured at 625 nm using the sodium phenolate–sodium hypochlorite colorimetric method Sucrase activity (S-SC) was assessed by quantifying the reducing sugars released from sucrose incubation using the 3,5-dinitrosalicylic acid (DNS) method, with absorbance measured at 540 nm. Polyphenol oxidase activity (S-PPO) was measured by monitoring the formation of quinones at 420 nm during the oxidation of *o*-diphenol. Acid phosphatase activity (S-ACP) was determined by measuring the *p*-nitrophenol released from disodium *p*-nitrophenyl phosphate at 400 nm [36]. The total phenolic content of the soil samples was assayed using the Folin–Ciocalteu method [37].

#### 2.4.4. Analysis of Microbial Diversity in Rhizosphere Soil of Patchouli

The microbial communities in patchouli rhizosphere soil were characterized by Illumina high-throughput sequencing. Genomic DNA was first extracted from the soil samples using the FastDNA^®^ SPIN Kit for Soil, with quality verified by 1% agarose gel electrophoresis. Subsequently, the V3-V4 region of the bacterial 16S rRNA gene and the fungal ITS1 region were amplified with primer pairs 341F/805R and ITS1F/ITS2, respectively. The amplicon libraries were constructed and sequenced on the Illumina NovaSeq^TM^ 6000 platform by LC Bio Technology Co., Ltd. (Hangzhou, China).

Following quality control (filtering, paired-end merging, and chimera removal), a total of 893,764 bacterial 16S rRNA and 1,096,330 fungal ITS high-quality sequences were obtained. The average base quality scores (Q20/Q30) were 96.6%/90.7% for bacteria and 99.1%/97.4% for fungi. These sequences were clustered into amplicon sequence variants (ASVs), yielding 25,315 bacterial and 15,323 fungal ASVs.

To assess sequencing completeness, Good’s coverage was calculated for each sample. For bacterial communities, coverage values ranged from 0.9983 to 0.9999, while fungal communities exhibited even higher coverage ranging from 0.9997 to 1.0000. These exceptionally high coverage values confirm that our sequencing depth was sufficient to comprehensively capture the microbial diversity present in each sample. To standardize sequencing depth across samples, all data were rarefied to 50,000 sequences per sample for bacteria and 68,000 for fungi, retaining all samples in both kingdoms. Relative abundance was calculated as (taxon sequences/total rarefied sequences) × 100% to describe community composition.

Based on the rarefied ASV table, we assessed taxonomic composition, alpha diversity, and differential abundance. Beta-diversity was evaluated using Weighted Unifrac distances, and community differences among treatments were tested with PERMANOVA (999 permutations). The raw sequencing data of 16S rRNA and ITS amplicons generated in this study have been deposited in the NCBI Sequence Read Archive (SRA) under BioProject accession numbers PRJNA1367888 (for bacterial communities) and PRJNA1367901 (for fungal communities).

### 2.5. Statistical Analysis

Experimental data were processed and statistically analyzed using Microsoft Excel 2016 and SPSS 27.0 (IBM Corp., Armonk, NY, USA). All data are presented as the mean ± standard deviation (SD) of three replicates (*n* = 3). The significance of differences among the treatment groups was determined by one-way analysis of variance (ANOVA). When the ANOVA indicated a significant main effect (*p* < 0.05), Duncan’s new multiple range test was applied for post hoc multiple comparisons. A significance level of *p* < 0.05 was used for all statistical tests. In tables and figures, different lowercase letters indicate statistically significant differences among treatments. Data visualization was performed using OriginPro 9.0 (OriginLab, Northampton, MA, USA) and R language (version 4.3.2; R Core Team, Vienna, Austria, 2023). Redundancy analysis (RDA) was conducted using Canoco 5.0 (Microcomputer Power, Ithaca, NY, USA).

## 3. Results

### 3.1. Mechanism of Phenolic Acid Removal by BC-UP Composite Particles

The phenolic acid removal mechanism of the BC-UP composite particle was investigated through batch degradation and EPR analysis. Using an initial *p*-CA concentration of 100 mg/L and a BC-UP composite dosage of 10 g/L, the degradation efficiency reached 93.6% within 180 min in the presence of Fe^2+^, significantly outperforming biochar (BC, 40.4%) or urea peroxide (UP, 48.2%) alone. The removal rate decreased to 61.8% without Fe^2+^, indicating the critical and synergistic partnership between Fe^2+^ and BC-UP in the degradation process. In a system with 2 g/L lateritic red soil, BC-UP maintained a high removal rate of 85.6%, vastly superior to the negligible removal (0.3%) by soil alone, underscoring the composite’s effectiveness in a soil environment.

As shown in Figure S2, EPR analysis identified •O_2_^−^, •OH, and ^1^O_2_ in the BC-UP+Fe^2+^ system, indicating a synergistic multi-radical oxidation system. No significant signals were detected with BC+Fe^2+^, confirming its adsorption-based mechanism. The UP+Fe^2+^ system produced a dominant •OH signal, with weaker •O_2_^−^ and ^1^O_2_ signals than BC-UP. Signals of •OH and •O_2_^−^ were also detected in the BC-UP+Soil system, confirming radical generation persists in soil. The BC-UP composite thus removes phenolic acids in soil via a combined mechanism of adsorption and multi-radical oxidation.

### 3.2. Effects of Different Exogenous Additives on Growth and Quality Indicators of Patchouli Cuttings

**Figure 1:**
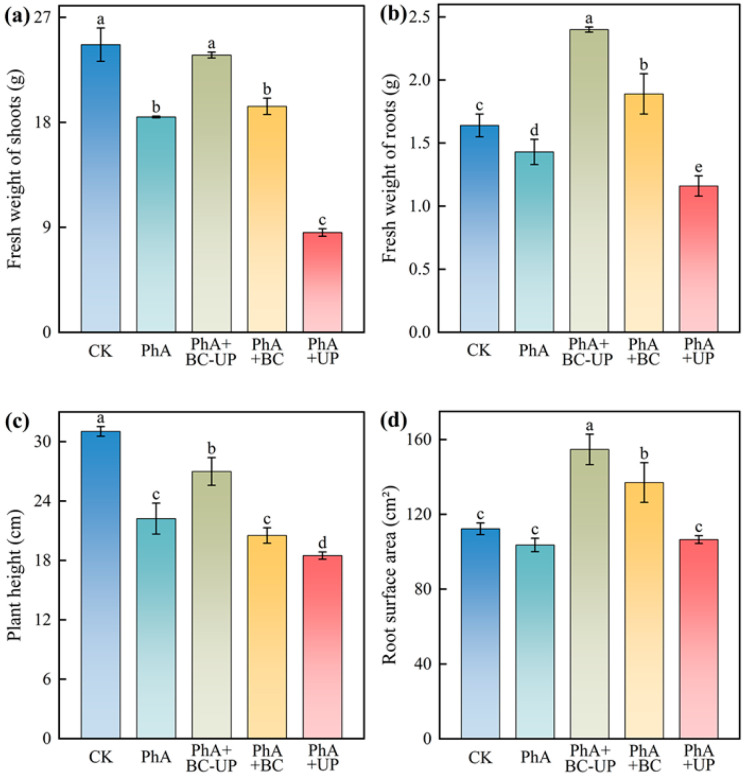
Growth indices of patchouli cuttings under different exogenous additive treatments. (**a**) Fresh weight of shoots, (**b**) Fresh weight of roots, (**c**) Plant height, (**d**) Root surface area. Data are presented as the mean ± SD (*n* = 3). Different lowercase letters above the bars indicate statistically significant differences among treatments as determined by one-way ANOVA followed by Duncan’s new multiple range test (*p* < 0.05).

As shown in Figure 1, phenolic acid treatment (PhA) significantly inhibited the growth of patchouli cuttings compared to the control (CK), reducing shoot fresh weight, root fresh weight, plant height, and root surface area by 25.1%, 12.8%, 28.4%, and 7.7% (*p* < 0.05), respectively. These results demonstrated that exogenous phenolic acids exerted a pronounced negative effect on patchouli plant growth.

Application of the biochar-urea peroxide composite (BC-UP) reversed this inhibition, significantly increasing these growth parameters by 28.8%, 67.8%, 21.4%, and 49.3% (*p* < 0.05) compared to the PhA treatment. Conversely, the PhA+UP treatment exacerbated the reduction in shoot fresh weight, root fresh weight, and plant height by 53.6%, 18.9%, and 16.8% (*p* < 0.05), respectively.

Furthermore, the PhA+BC-UP treatment outperformed the individual PhA+BC and PhA+UP treatments, increasing shoot fresh weight by 22.7% and 177.7%, root fresh weight by 27.0% and 106.9%, plant height by 31.5% and 46.0%, and root surface area by 12.9% and 45.3% (*p* < 0.05), respectively. This demonstrates the superior efficacy of the combined BC-UP conditioner in alleviating phenolic acid-induced growth inhibition.

**Figure 2:**
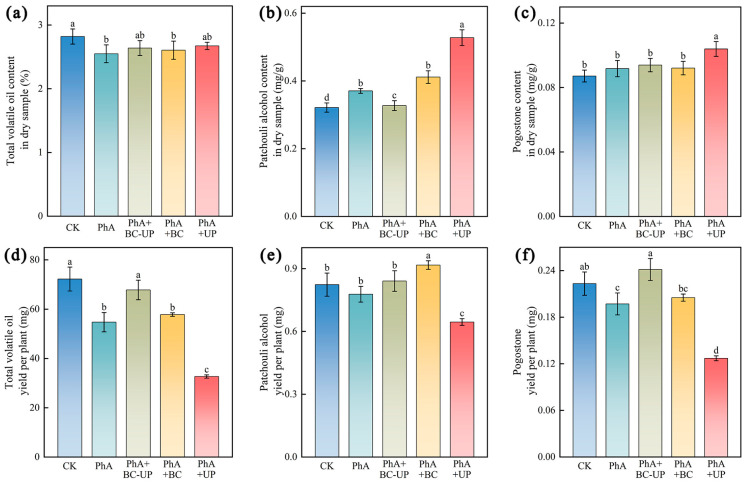
Effects of exogenous additives on quality indicators of active compounds in patchouli cuttings: in dry matter (**a**–**c**): (**a**) total volatile oil content, (**b**) patchouli alcohol content, (**c**) pogostone content; and quality indicators of fresh patchouli plants per individual plant (**d**–**f**): (**d**) total volatile oil yield, (**e**) patchouli alcohol yield, (**f**) pogostone yield per plant. Data are presented as the mean ± SD (*n* = 3). Different lowercase letters above the bars indicate statistically significant differences among treatments as determined by one-way ANOVA followed by Duncan’s new multiple range test (*p* < 0.05).

The impact of exogenous additives on key pharmacological components in patchouli was assessed in both dried and fresh samples (Figure 2).

In dried samples (Figure 2a–c), the phenolic acid (PhA) treatment reduced the total volatile oil content by 9.6% relative to CK (*p* < 0.05). This suppression was alleviated by PhA+BC-UP, PhA+BC, and PhA+UP treatments, which increased oil content by 2.2% to 4.9% compared to PhA. Conversely, PhA increased patchouli alcohol by 15.3% (*p* < 0.05), an effect further enhanced by PhA+UP, which resulted in the highest level (42.4% higher than PhA; *p* < 0.05). All treatments elevated the pogostone content over the CK: the PhA, PhA+BC-UP, PhA+BC, and PhA+UP treatments resulted in increases of 5.3%, 7.8%, 5.6%, and 19.3%, respectively.

In fresh shoots per plant (Figure 2d–f), PhA significantly reduced biomass and the yield of all components, decreasing total volatile oil, patchouli alcohol, and pogostone by 24.2%, 5.5%, and 11.7%, respectively (*p* < 0.05 for oil and pogostone). BC-containing particles counteracted this reduction: PhA+BC-UP increased these components by 23.9%, 8.1%, and 22.5%, while PhA+BC increased them by 5.6%, 17.9%, and 4.1%, respectively, compared to PhA. In contrast, PhA+UP severely inhibited biomass, resulting in the lowest per-plant yield of active components. Thus, BC-UP particles achieved a superior balance in enhancing the comprehensive yield of active compounds.

### 3.3. Effects of Different Exogenous Additives on Physicochemical Properties and Enzyme Activity of Patchouli Rhizosphere Soil

As presented in Table 1, exogenous phenolic acids (PhA) increased the total phenolic acid content in the rhizosphere soil by 63.7% compared to CK. This was significantly reversed by the PhA+BC-UP, PhA+BC, and PhA+UP treatments, which reduced the content by 37.3%, 31.8%, and 18.9%, respectively, with the composite particles (PhA+BC-UP) showing the greatest reduction.

The PhA treatment also significantly decreased soil pH and organic matter relative to CK, while alkali-hydrolyzable nitrogen (AN), available phosphorus (AP), and available potassium (AK) showed no significant differences. Application of BC-UP particles increased pH by 0.15 units and significantly raised organic matter and AN by 10.1% and 37.7% (*p* < 0.05) over the PhA treatment, with AP and AK also showing non-significant increases. Compared to the PhA treatment, the PhA+BC treatment elevated pH by 0.44 units and significantly enhanced organic matter, AN, and AK by 21.9%, 28.2%, and 14.8% (*p* < 0.05), respectively, though AP decreased by 6.5%, potentially due to biochar-induced pH elevation. Conversely, PhA+UP significantly decreased pH by 0.17 units and reduced organic matter by 41.2%, while substantially increasing AN and AK by 75.9% and 16.1%; AP remained unchanged.

**Figure 3:**
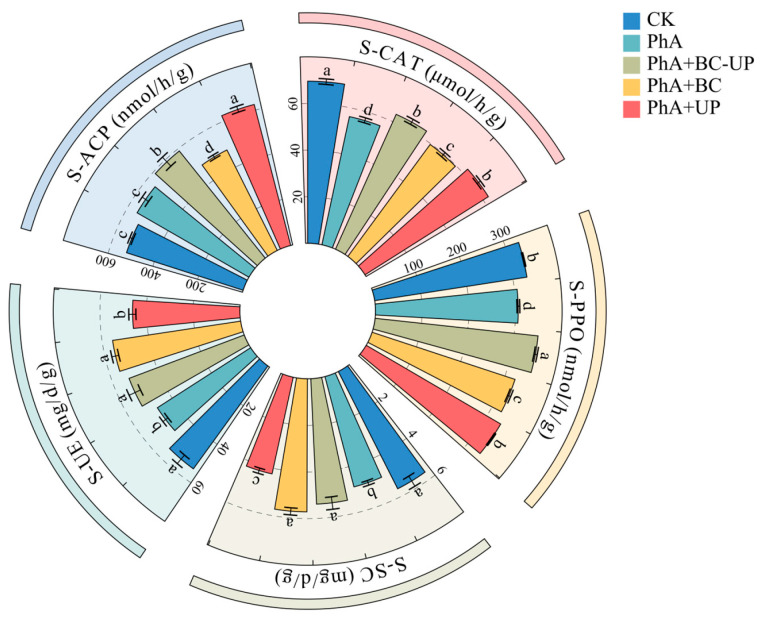
Rhizosphere soil enzyme activities of patchouli cuttings under different exogenous additive treatments. Soil catalase activity (S-CAT), Soil polyphenol oxidase activity (S-PPO), Soil sucrase activity (S-SC), Soil urease activity (S-UE), Soil acid phosphatase activity (S-ACP). Data are presented as the mean ± SD (*n* = 3). Different lowercase letters above the bars indicate statistically significant differences among treatments as determined by one-way ANOVA followed by Duncan’s new multiple range test (*p* < 0.05).

As shown in Figure 3, exogenous phenolic acids (PhA) significantly suppressed the activity of most soil enzymes compared to CK, reducing catalase, polyphenol oxidase, sucrase, and urease by 7.3% to 19.1% while increasing acid phosphatase.

This broad inhibition was effectively alleviated by the BC-UP composite particles, which significantly increased the activities of all five enzymes by 6.5% to 14.9% relative to the PhA treatment. Similarly, biochar (BC) alone also enhanced most enzyme activities, with sucrase and urease showing the most pronounced increases (16.6% and 16.7%), followed by catalase (6.0%) and polyphenol oxidase (5.5%). However, the rise in soil pH from biochar significantly reduced acid phosphatase activity. The PhA+UP treatment increased catalase, polyphenol oxidase, and acid phosphatase by 9.3% to 12.9% but was less effective for other enzymes. Cross-comparison confirmed BC-UP’s superior overall efficacy, as BC exhibited significantly lower activities in catalase, polyphenol oxidase, and acid phosphatase, while UP showed higher acid phosphatase but lower activities in the other four enzymes. In summary, BC-UP provided the most balanced and comprehensive alleviation of phenolic acid-induced suppression of soil enzyme activity.

### 3.4. Effects of Different Exogenous Additives on Microbial Diversity in the Rhizosphere Soil of Patchouli

#### 3.4.1. Alpha Diversity Analysis of Microbial Communities in the Rhizosphere Soil of Patchouli Treated with Different Exogenous Additives

**Figure 4:**
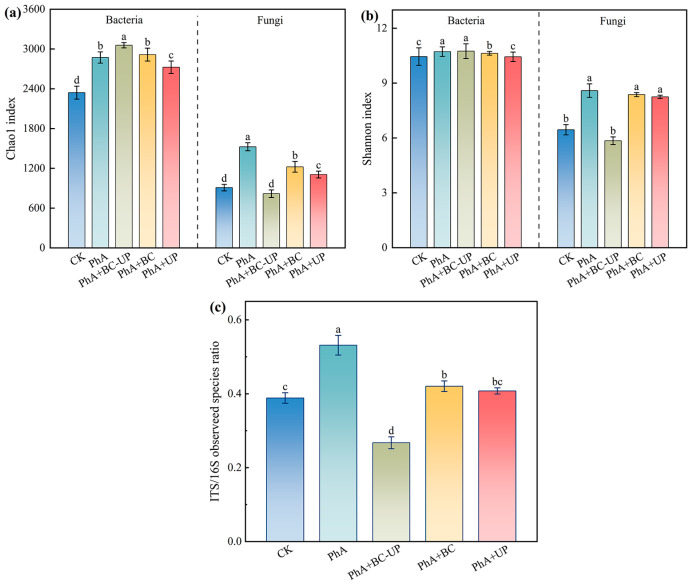
(**a**) Bacterial and fungal Chao1 index, (**b**) Shannon index, (**c**) fungal-to-bacterial (ITS/16S) OTU ratio in the rhizosphere soil of patchouli cuttings under different exogenous additive treatments. Data are presented as the mean ± SD (*n* = 3). Different lowercase letters above the bars indicate statistically significant differences among treatments as determined by one-way ANOVA followed by Duncan’s new multiple range test (*p* < 0.05).

Alpha diversity analysis (Figure 4) revealed that the phenolic acid treatment (PhA) significantly promoted bacterial richness and evenness compared to CK, increasing the Chao1 and Shannon indices by 22.6% (*p* < 0.05) and 2.6%, respectively. Compared to PhA, the addition of BC-UP and BC particles further increased bacterial richness (Chao1 index), with PhA+BC-UP showing the greatest rise of 6.4% (*p* < 0.05). Regarding diversity (Shannon index), only PhA+BC-UP showed a marginal increase (0.3%), while PhA+BC and PhA+UP led to slight decreases.

Compared to the control (CK), the PhA treatment profoundly enhanced fungal diversity, increasing the Chao1 and Shannon indices by 67.8% and 33.2% (*p* < 0.05) versus CK. This effect was reversed by BC-UP particles, which significantly reduced these indices by 46.4% and 31.9%, respectively, compared to PhA. PhA+BC and PhA+UP also slightly lowered fungal diversity.

Furthermore, PhA increased the fungal-to-bacterial OTU ratio (ITS/16S) by 36.7% versus CK (Figure 4c), indicating a shift toward a fungal-dominated state [38]. In contrast, all particle additions (BC-UP, BC, UP) significantly reduced this ratio under phenolic acid stress by 20.9% to 49.7% compared to PhA, with the BC-UP treatment showing the greatest reduction (49.7%). Thus, the BC-UP composite effectively mitigated the phenolic acid-induced microbial community shift by enhancing bacterial diversity, suppressing fungal dominance, and restoring community balance.

#### 3.4.2. Beta Diversity Analysis of Microbial Communities in the Patchouli Rhizosphere Soil Treated with Different Exogenous Additives

Principal Component Analysis (PCA) revealed distinct shifts in the rhizosphere soil microbial community structure under different treatments. For bacterial communities (Figure S3a), the first two principal components (PCA1 and PCA2) explained 54.03% and 13.30% of the variation, respectively (cumulative 67.33%). The PhA treatment samples were clearly separated from the clustered CK control, indicating community restructuring. Samples from PhA+BC-UP, PhA+BC, and PhA+UP treatments showed varying degrees of separation from PhA, confirming their mitigating effects.

A similar pattern was observed for fungal communities (Figure S3b), with PCA1 and PCA2 explaining 61.58% and 14.18% of the variance, respectively. The CK group was distinctly separated, confirming PhA-induced restructuring. The PhA+BC-UP replicates formed a distinct cluster away from PhA, while PhA+BC and PhA+UP remained closer, suggesting BC-UP was more effective in altering the fungal community.

To statistically validate the separations of the treatments, PERMANOVA was performed based on the Weighted Unifrac distance matrix. The analysis revealed that treatments significantly shaped both bacterial (R^2^ = 0.693, *F* = 5.644, *p* = 0.001) and fungal (R^2^ = 0.451, *F* = 2.050, *p* = 0.001) communities, confirming the observed differences were robust and not due to chance.

#### 3.4.3. Effects of Different Treatments on Bacterial and Fungal Community Structures in Rhizosphere Soil

**Figure 5:**
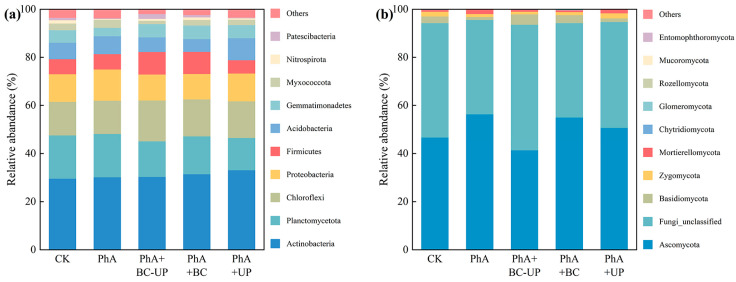
Phylum-level community structure of bacteria (**a**) and fungi (**b**) in patchouli rhizosphere soil under different treatment.

The rhizosphere soil microbial community composition under different treatments is shown in Figure 5. At the bacterial phylum level (Figure 5a), seven major phyla (relative abundance >2%) constituted 81.7–93.9% of the community: Actinobacteriota (29.6–33.1%), Planctomycetota (13.4–18.0%), Chloroflexi (13.8–17.0%), Proteobacteria (10.6–13.0%), Firmicutes (5.5–9.4%), Acidobacteriota (5.3–9.2%), and Gemmatimonadota (3.5–5.7%). Actinobacteriota was consistently dominant. Compared to the PhA treatment, both PhA+BC-UP and PhA+BC increased the abundance of Firmicutes while reducing Acidobacteriota.

At the fungal phylum level (Figure 5b), five phyla (relative abundance >1%) accounted for 99.66–99.96% of the community: Ascomycota (41.4–56.3%), unclassified Fungi (39.3–52.2%), Basidiomycota (1.5–4.3%), Zygomycota (1.1–2.1%), and Mortierellomycota (0.7–1.9%). Phenolic acid stress (PhA) increased the abundances of Ascomycota and Mortierellomycota relative to CK. All soil conditioner treatments (PhA+BC-UP, PhA+BC, PhA+UP) significantly reduced Ascomycota abundance and increased Basidiomycota abundance compared to PhA.

Fungal genus-level analysis (Figure S4) revealed that PhA significantly increased the relative abundance of *Humicola* and *Mortierella* compared to CK. In contrast, the BC-UP application markedly suppressed several genera, including *Aspergillus*, *Gibberella*, *Haematonectria*, *Microascus*, and *Mortierella*, reducing their abundances by 30.8% to 55.8% relative to PhA (*p* < 0.05).

#### 3.4.4. Screening of Biomarks in Patchouli Rhizosphere Soil Under Exogenous Phenolic Acid and Composite Particle Treatments

LefSe analysis (LDA effect size analysis) was used, with LDA > 3.5 and *p* < 0.05, to identify biomarkers of soil microbial under three treatments, including CK, PhA treatment, and PhA+BC-UP treatment. The comparison between the CK and PhA treatments (Figure S5) revealed a significant enrichment of the bacterial phyla Gemmatimonadota and Nitrospirota in the CK group. This finding suggests that exogenous phenolic acid stress substantially inhibited the abundance of these bacteria in the patchouli rhizosphere soil. A distinct shift in the microbial community was observed when comparing the PhA and PhA+BC-UP treatments (Figure S6). The PhA treatment was characterized by significant enrichment of Planctomycetota, Proteobacteria, Myxococcota, and Methylomirabilota. In contrast, the PhA+BC-UP treatment showed a marked increase in Firmicutes, Gemmatimonadota, and Patescibacteria.

Fungal community analysis provided further insights (Figures S7 and S8). The phylum Ascomycota was significantly more abundant in the PhA treatment compared to both the CK and PhA+BC-UP treatments, indicating a specific fungal response to phenolic acid stress. Conversely, Basidiomycota was significantly enriched in the CK and PhA+BC-UP treatments. This suggests that the PhA+BC-UP treatment helped in restoring a fungal community composition more similar to the untreated control.

**Figure 6:**
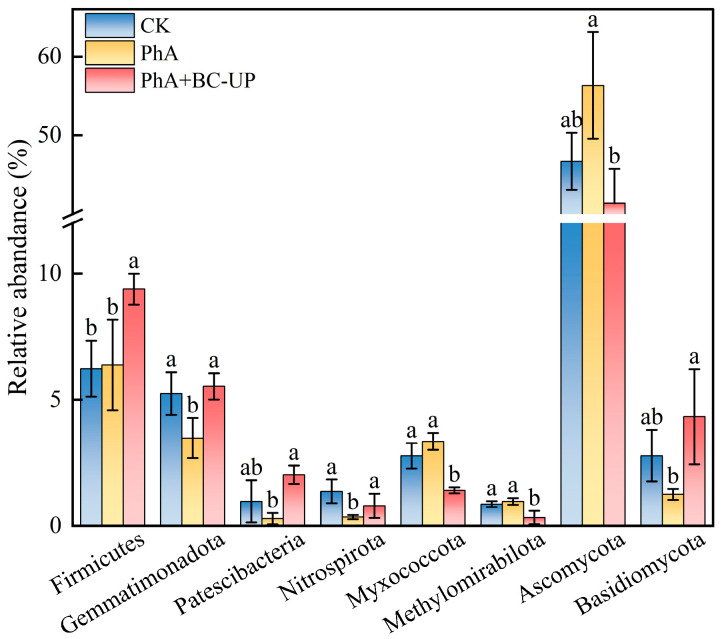
Relative abundance of signature biomarkers in rhizosphere soil under CK, PhA, PhA+BC-UP treatment. Data are presented as the mean ± SD (*n* = 3). Different lowercase letters above the bars indicate statistically significant differences among treatments as determined by one-way ANOVA followed by Duncan’s new multiple range test (*p* < 0.05).

ANOVA revealed significant differences (*p* < 0.05) in the relative abundance of six bacterial and two fungal species among the CK, PhA, and PhA+BC-UP treatments (Figure 6). Compared to the PhA, PhA+BC-UP significantly increased the relative abundance of Firmicutes, Gemmatimonadota, Patescibacteria, and Nitrospirota by 0.47-, 0.58-, 6.12-, and 1.29-fold, respectively. Conversely, the abundances of Myxococcota and Methylomirabilota were significantly reduced by 58.01% and 66.43%. For fungi, the PhA treatment increased Ascomycota and decreased Basidiomycota relative to CK. Notably, the PhA+BC-UP treatment reversed this trend, reducing Ascomycota by 26.55% and increasing Basidiomycota by 2.47-fold compared to PhA (*p* < 0.05).

### 3.5. Correlation Analysis of Patchouli Biomass with Rhizosphere Soil Environmental Factors and Microbial Community Abundance

**Figure 7:**
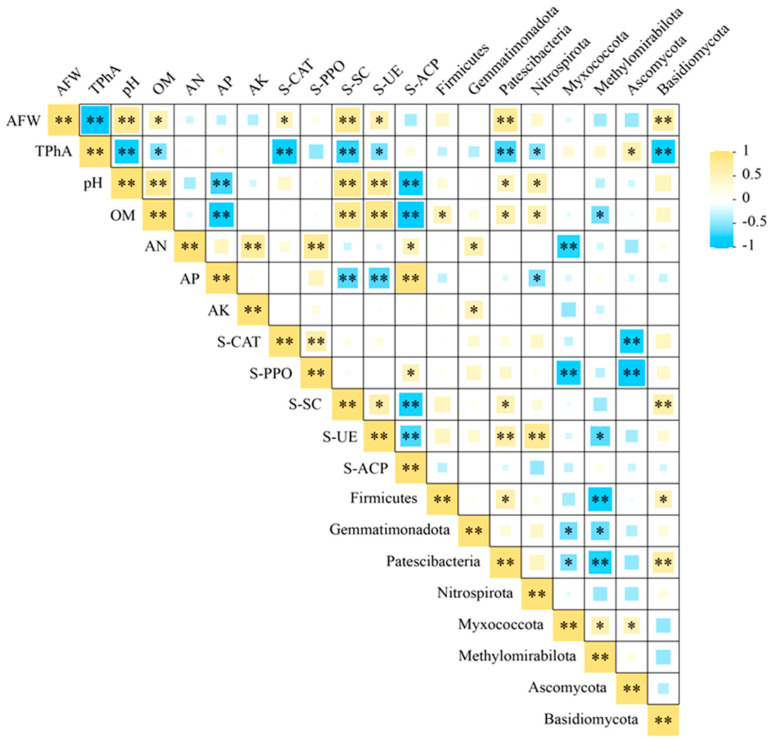
Correlation heatmap between the aboveground fresh weight of patchouli and key soil environmental factors. Aboveground fresh weight of patchouli (AFW), Total phenolic acid in soil (TPhA), Organic matter content (OM), Alkaline hydrolyzable nitrogen (AN), Available phosphorus (AP), Available potassium (AK). Soil catalase activity (S-CAT), Soil polyphenol oxidase activity (S-PPO), Soil sucrase activity (S-SC), Soil urease activity (S-UE), Soil acid phosphatase activity (S-ACP). *p* < 0.05 (*); *p* < 0.01 (**).

To identify potential factors affecting patchouli yield, Spearman correlation analysis was employed to assess the relationships between aboveground fresh weight and rhizosphere soil properties, enzyme activities, and key microbial communities. As illustrated in Figure 7, the biomass was strongly negatively correlated with total phenolic acids (r = −0.892). Conversely, it was significantly positively correlated with several factors: soil pH, sucrase activity, and the relative abundances of Patescibacteria and Basidiomycota (*p* < 0.01); as well as with organic matter, catalase, urease, and Nitrospirota (*p* < 0.05).

Further analysis showed that rhizosphere phenolic acids were positively correlated with Ascomycota abundance (*p* < 0.01), but negatively correlated with soil pH, catalase, urease activity, the relative abundances of Patescibacteria, Nitrospirota, and Basidiomycota (*p* < 0.01), as well as with polyphenol oxidase and sucrase activities (*p* < 0.05).

**Figure 8:**
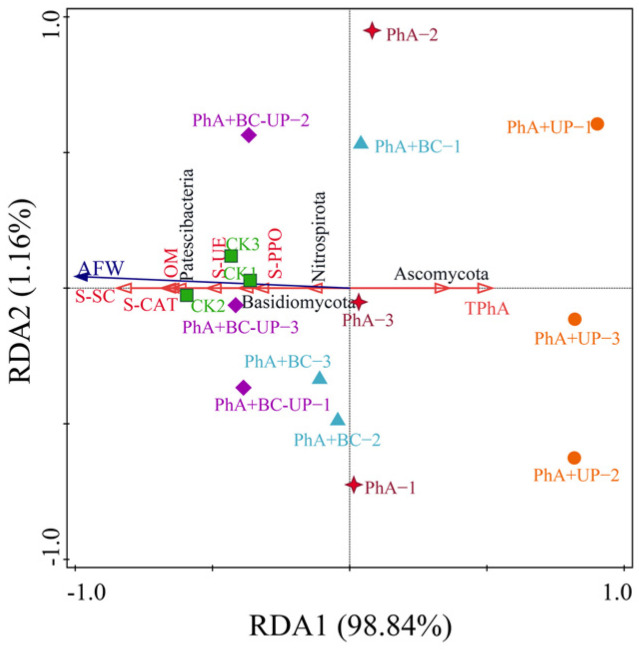
Redundancy analysis of the aboveground fresh weight of patchouli and key soil environmental factors. Aboveground fresh weight of patchouli (AFW), Total phenolic acid in soil (TPhA), Organic matter content (OM), Soil catalase activity (S-CAT), Soil polyphenol oxidase activity (S-PPO), Soil sucrase activity (S-SC), Soil urease activity (S-UE).

Redundancy analysis (RDA) was performed to elucidate the interactions between aboveground biomass and 11 key factors previously identified as significantly associated with it and with phenolic acids (Figure 8). The first two RDA axes effectively explained the variation (98.84% and 1.16%, respectively). The analysis showed that soil sucrase (S-SC) was the strongest predictor (71.7%, *p* = 0.002), followed by total phenolic acids (TPhA, 10.5%, *p* = 0.004), Ascomycota abundance (9.2%, *p* = 0.022), and catalase (S-CAT, 3.2%, *p* = 0.012); other factors were non-significant.

The RDA plot revealed a clear grouping: the PhA treatment was associated with the positive side of the first axis, showing positive correlations with TPhA and Ascomycota. In contrast, both the PhA+BC-UP treatment and the CK control clustered together in a region characterized by positive correlations with S-SC and S-CAT and negative correlations with TPhA and Ascomycota, indicating an antagonistic regulatory effect between phenolic acids and the BC-UP composite.

## 4. Discussion

### 4.1. From Phenolic Acid Stress-Induced Inhibition to Conditioner-Mediated Alleviation: Growth and Quality Responses in Patchouli

The significant growth inhibition observed in patchouli cuttings under exogenous phenolic acid (PhA) stress, which evidenced by reduced biomass and impaired root morphology, is consistent with reports across other medicinal plants, such as *Pinellia ternata* and *Salvia miltiorrhiza* [6,39]. This consensus underscores a generalized phytotoxic mechanism wherein phenolic acids disrupt physiological processes, compromise cell membrane integrity, and induce oxidative stress [40,41]. The application of biochar (BC) alone partially mitigated this stress, improving biomass and root architecture, a finding aligned with studies on *Bletilla striata* and celery continuous cropping [42,43]. This mitigation is primarily attributed to the adsorption of phenolic acids by BC’s porous structure and the subsequent restoration of the rhizosphere microbiome [44,45]. However, the novel biochar–urea peroxide composite (BC-UP) demonstrated superior efficacy, driving significant additional gains in shoot (12.3%) and root (18.7%) fresh weight compared to BC alone. This enhancement stems from a synergistic “adsorption–oxidation” mechanism: BC concentrates phenolic acids, while the slowly released H_2_O_2_ from UP generates free radicals for their oxidative degradation [12]. Beyond direct detoxification, the mild oxidative stress induced by BC-UP may act as a signaling cue, potentially contributing to the observed boost in root growth and overall plant vigor.

Phenolic acid stress triggered a “stress-induced” defense response in patchouli, increasing the relativecontent of key active compounds (volatile oil, patchouli alcohol and pogostone) [46]. Nevertheless, the substantial decrease in biomass led to an overall reduction in the total yield of these valuable components. The BC-UP composite uniquely addressed this compromise. It not only restored total volatile oil accumulation to levels comparable to the non-stress control but also significantly increased pogostone content by 26.4% compared to the PhA-stressed group. This indicates that BC-UP achieves a dual benefit: it effectively neutralizes the phytotoxic stress while simultaneously creating a condition that may promote the biosynthesis and accumulation of specific pharmacologically active compounds. Consequently, the BC-UP composite achieves the triple effect of detoxifying the soil, promoting plant growth, and improving medicinal quality.

### 4.2. Soil Property and Enzyme Response to Phenolic Acid Stress and Conditioner Remediation

Exogenous phenolic acid (PhA) stress significantly degraded the rhizosphere soil environment of patchouli, inducing soil acidification, nutrient imbalance (e.g., reductions in organic matter and alkali-hydrolyzable nitrogen), and a marked accumulation of total phenolic acids. This aligns with the soil degradation patterns commonly observed in continuous cropping systems [47,48]. Furthermore, PhA stress broadly suppressed the activities of key enzymes involved in C and N cycling, such as catalase, polyphenol oxidase, sucrase, and urease, thereby impairing rhizosphere ecological functioning [49]. The slight increase in acid phosphatase activity under PhA treatment may be a specific response to the acidified soil environment, reflecting the functional adaptation of soil enzymes to stress.

In contrast, the BC-UP composite particles demonstrated a superior ameliorative effect by integrating the individual advantages of BC and UP. Biochar (BC) improved the soil microenvironment through its porous structure and functional groups, which adsorb phenolic acids and enhance nutrient retention, thereby promoting the activities of sucrase, urease, and catalase [50,51,52]. Urea peroxide (UP) efficiently degraded phenolic acids Via oxidative free radicals. However, its decomposition led to soil acidification, which, while stimulating acid phosphatase activity, exerted an oxidative inhibitory effect on other enzymes. The BC-UP composite effectively mitigated this acidification Via the alkaline functional groups of BC, while the confinement of UP-derived free radicals within biochar micropores enabled a more sustained and controllable oxidation of phenolic pollutants [53,54]. Consequently, BC-UP outperformed the individual BC or UP treatments in enhancing key enzyme activities and achieving the most significant reduction in soil phenolic acid content.

### 4.3. Shaping of Rhizosphere Microbial Community Structure by Phenolic Acid Stress and BC-UP Remediation

#### 4.3.1. BC-UP Particles Reverse the Microbial Community “Fungalization” Trend

Exogenous phenolic acids (PhA) altered the patchouli rhizosphere microbiome by increasing the richness of both bacterial and fungal communities and elevating the fungi-to-bacteria ratio, indicating a shift toward a fungal-dominated state [55]. This “fungalization” trend, often associated with acidic environments and potentially pathogenic proliferation, represents a risk in degraded soils [56]. In contrast, the BC-UP composite particles counteracted this shift. By effectively removing phenolic acids and alleviating soil acidification, BC-UP created a microenvironment more favorable for bacterial growth. This resulted in enhanced bacterial diversity, suppression of fungal dominance, and a consequent reduction in the ITS/16S OTU ratio. These findings demonstrate that BC-UP fundamentally alters the bacterial-fungal competitive balance through rapid improvement of the soil chemical environment, thereby mitigating the ecological risks induced by phenolic acid accumulation.

#### 4.3.2. BC-UP Particles Reshape the Bacterial Community Structure: Enrichment of Bacterial Phyla with Putative Beneficial Functions

At the bacterial phylum level, phenolic acid stress enriched acid-tolerant taxa such as Acidobacteria, consistent with patterns of soil acidification [57]. The BC-UP amendment counteracted this shift by reducing the relative abundance of oligotrophic and acidophilic groups while enriching several bacterial phyla commonly associated with beneficial soil functions. Specifically, the increased abundances of Actinobacteria and Firmicutes, groups known for degrading recalcitrant organic compounds like lignin and phenolic acids, which likely contributed directly to stress alleviation and nutrient cycling [58,59,60]. Chloroflexi and Gemmatimonadota serve as biological markers of soil health and facilitate carbon and nitrogen cycling [61]; and Nitrospirota enhance nitrification by converting nitrite to nitrate [62]. The pore structure and soluble organic carbon provided by the biochar component likely created favorable niches for these copiotrophic bacteria. Consequently, BC-UP not only established a more neutral microenvironment with a sustained nutrient supply but also shifted the bacterial community by reducing taxa associated with acidic, oligotrophic conditions and enriching phyla that are commonly linked to improved soil functioning.

#### 4.3.3. Fungal Community Shift Induced by BC-UP: Suppression of Pathogen-Associated Phyla

The fungal community exhibited a more pronounced response to phenolic acid (PhA) stress and BC-UP amendment compared to the bacterial community. PhA treatment significantly enriched phyla such as Ascomycota and Mortierellomycota, which harbor numerous documented plant pathogenic fungi and are frequently linked to an elevated risk of soil-borne diseases [63]. In contrast, BC-UP amendment effectively reduced the relative abundance of these phyla while substantially increasing the proportion of Basidiomycota, a group recognized for its saprophytic capabilities and biocontrol potential, often contributing to organic matter decomposition and pathogen antagonism [64]. This shift aligns with prior findings on biochar’s capacity to suppress pathogenic fungi [65].

At the genus level, the PhA treatment enriched genera like *Humicola* and *Mortierella*. While *Mortierella* contains some plant-growth-promoting species, its over-proliferation under stress might indicate an imbalanced environment. Notably, the BC-UP treatment significantly suppressed the relative abundance of several genera that include known pathogenic species, such as *Aspergillus* (which encompasses mycotoxin-producing species), *Gibberella* (a genus containing major causal agents of *Fusarium* wilt), and *Haematonectria* (a genus comprising plant pathogen) [66]. Thus, BC-UP appears to regulate the fungal community structure by reducing the prevalence of taxa that harbor potential pathogens while favoring putatively beneficial saprophytes, thereby potentially enhancing the biological resistance of the soil micro-ecosystem.

#### 4.3.4. Integrated Analysis Reveals Synergistic Microecological Regulation by BC-UP Particles in Alleviating Autotoxicity

Both Spearman correlation analysis and Redundancy Analysis (RDA) revealed that patchouli shoot biomass was positively associated with soil pH, sucrase and catalase activities, and the abundance of Patescibacteria and Basidiomycota, but negatively correlated with total phenolic acid content and Ascomycota abundance. Phenolic acid accumulation was linked to the suppression of key nitrogen-cycling bacteria (e.g., Nitrospirota) and enzyme activities, likely impairing nutrient turnover and inducing oxidative stress. In contrast, the BC-UP composite counteracted these effects by simultaneously elevating soil pH, enhancing enzyme activities, reducing phenolic acid levels, and promoting potential beneficial bacterial taxa while suppressing potentially pathogenic fungi. This integrated improvement in both chemical and biological soil properties supports a conceptual model in which BC-UP alleviates autotoxicity through multi-faceted microecological regulation. This pattern, in which soil amendments regulate the soil microbial community structure by modifying environmental factors such as pH and enzyme activities, has also been confirmed by Xu et al. [67]. Therefore, these findings suggest that BC-UP composite particles alleviate phenolic acid stress by synergistically modulating the physicochemical and biological properties of the rhizosphere soil through multiple factors.

Beyond mechanistic efficacy, the BC-UP strategy aligns with circular economy and sustainable agriculture principles. The biochar component can be produced from agricultural wastes, embodying a “waste-to-resource” approach. Its in-situapplication offers a sustainable alternative to energy-intensive soil replacement, while urea peroxide serves a dual role as both oxidant and nitrogen fertilizer, enhancing nutrient use efficiency. Therefore, the BC-UP strategy not only provides a viable remedy for continuous cropping obstacles, but also provides an environmentally coherent solution that contributes to closing resource loops in agricultural systems.

## 5. Conclusions

This study demonstrates that biochar–urea peroxide composite particles (BC-UP) effectively alleviate phenolic acid stress in *Pogostemon cablin* under controlled conditions. The application of BC-UP significantly enhanced patchouli growth, bioactive compound accumulation, and improved key soil properties while reducing rhizosphere phenolic acid content. Mechanistically, BC-UP functioned through the direct degradation of allelochemicals, enhancement of soil fertility and enzyme activities, and the reassembly of a beneficial root-associated microbiome.

Microbial community analysis revealed that BC-UP amendment notably enriched bacterial taxa such as Firmicutes and Nitrospirota, which are often associated with nutrient cycling and soil health. Concurrently, it suppressed the abundance of the Ascomycota phylum, which includes many known plant-pathogenic fungi. Redundancy analysis identified soil total phenolic acid content, Ascomycota abundance, sucrase activity, and catalase activity as key factors influencing patchouli biomass.

Despite these promising results, it is important to acknowledge the limitations of this study and the necessary future directions. The primary limitations include: (1) the controlled greenhouse conditions, which may not fully reflect field realities; (2) the use of only one variety of *Pogostemon cablin*, suggesting that results may vary across species; and (3) the unassessed long-term effects of BC-UP on continuous cropping systems and overall ecosystem sustainability. Based on these limitations, we propose the following future research projections: (1) conducting field-scale trials across diverse soil types and medicinal crops to validate efficacy; (2) exploring interactions between BC-UP and key agronomic factors (e.g., fertilization, irrigation); and (3) performing comprehensive economic and environmental assessments to evaluate the practical viability of BC-UP in sustainable agriculture.

## Figures and Tables

**Table 1:** Effects of different exogenous additive treatments on physicochemical properties of patchouli rhizosphere soil.

Treatment	Total Phenolic Acids (μg/g)	pH	Organic Matter (g/kg)	Alkali-Hydrolysable N (mg/kg)	Available P (mg/kg)	Available K (mg/kg)
CK	6.56 ± 0.12 d	6.36 ± 0.01 a	25.85 ± 0.58 b	90.76 ± 0.87 d	162.34 ± 1.42 b	83.94 ± 0.76 b
PhA	10.73 ± 0.49 a	5.51 ± 0.06 d	22.85 ± 0.57 c	87.51 ± 1.21 d	165.11 ± 1.69 ab	83.26 ± 1.36 b
PhA+BC-UP	6.73 ± 0.12 d	5.66 ± 0.07 c	25.12 ± 0.91 b	120.51 ± 1.65 b	166.72 ± 2.21 a	86.53 ± 2.39 b
PhA+BC	7.32 ± 0.09 c	5.95 ± 0.07 b	27.85 ± 0.32 a	112.17 ± 3.1 c	154.38 ± 0.87 c	95.57 ± 0.48 a
PhA+UP	8.70 ± 0.08 b	5.34 ± 0.08 e	21.40 ± 0.18 d	153.96 ± 3.05 a	168.58 ± 3.12 a	96.68 ± 3.14 a

Data are presented as mean ± SD (*n* = 3). Different lowercase letters within a column indicate significant differences between treatments according to Duncan’s multiple range test (*p* < 0.05).

## Data Availability

The original contributions presented in this study are included in the article/Supplementary Materials. Further inquiries can be directed to the corresponding authors.

## Supplementary Materials

The following supporting information can be downloaded at: https://www.mdpi.com/article/10.3390/microorganisms13122772/s1, Figure S1. SEM micrograph and FTIR spectra of BC-UP composite particles. Figure S2. Comparison of electron paramagnetic resonance (EPR) spectra of ROS trapped by DMPO and TEMP in different treatment systems. Figure S3: PCA of bacterial (a) and fungal (b) communities across different exogenous additive treatments; Figure S4: Fungal community structure at the genus level in patchouli rhizosphere soil across treatments; Figure S5: Bar plots of LDA scores from LEfSe analysis identifying differentially bacterial taxa in rhizosphere soil between CK and PhA treatments; Figure S6: Bar plots of LDA scores from LEfSe analysis identifying differentially bacterial taxa in rhizosphere soil between PhA and composite additive treatments; Figure S7: Bar plots of LDA scores from LEfSe analysis identifying differentially fungal taxa in rhizosphere soil between CK and PhA treatments; Figure S8: Bar plots of LDA scores from LEfSe analysis identifying differentially fungal taxa in rhizosphere soil between PhA and composite additive treatments. Table S1: Growth parameters of *Pogostemon cablin* under different addition amounts of BC-UP composite particles.

## References

[1] Phuwajaroanpong A., Chaniad P., Horata N., Muangchanburee S., Kaewdana K., Punsawad C. (2020). In vitro and In vivo antimalarial activities and toxicological assessment of *Pogostemon Cablin* (Blanco) Benth. J. Evid.-Based Integr. Med..

[2] Yan W., Cao S., Wu Y., Ye Z., Zhang C., Yao G., Yu J., Yang D., Zhang J. (2022). Integrated analysis of physiological, mRNA sequencing, and miRNA sequencing data reveals a specifc mechanism for the response to continuous cropping obstacles in *Pogostemon cablin* roots. Front. Plant Sci..

[3] Sun Y., Su Y., Hussain A., Xiong L., Li C., Zhang J., Meng Z., Dong Z., Yu G. (2023). Complete genome sequence of the *Pogostemon cablin* bacterial wilt pathogen *Ralstonia solanacearum* strain SY1. Genes Genom..

[4] Wang Y., Zhang Y., Jin H., Deng Z., Li Z., Mai Y., Li G., He H. (2019). A practical random mutagenesis system for Ralstonia solanacearum strains causing bacterial wilt of *Pogostemon cablin* using Tn5 transposon. World J. Microbiol. Biotechnol..

[5] He Z., Wang Y., Yan Y., Qin S., He H., Mao R., Liang Z. (2022). Dynamic analysis of physiological indices and transcriptome profiling revealing the mechanisms of the allelopathic effects of phenolic acids on *Pinellia ternata*. Front. Plant Sci..

[6] He Y., Zhang M., Zhou W., Ai L., You J., Liu H., You J., Wang H., Wassie M., Wang M. (2019). Transcriptome analysis reveals novel insights into the continuous cropping induced response in *Codonopsis tangshen*, a medicinal herb. Plant Physiol. Biochem..

[7] Zhang J., Fan S., Qin J., Dai J., Zhao F., Gao L., Lian X., Shang W., Xu X., Hu X. (2020). Changes in the microbiome in the soil of an American ginseng continuous plantation. Front. Plant Sci..

[8] Zhou Y., Liu Y., Li S., Yang Q. (2024). The combination of biochar and *Bacillus subtilis* biological agent reduced the relative abundance of pathogenic bacteria in the rhizosphere soil of *Panax notoginseng*. Microorganisms.

[9] Li L., Luo X., Liu J., Yao M., Liu Y., Ma Y., Huang L.u., Sun X. (2025). Promoting international acceptance of clinical studies about traditional Chinese medicine interventions. Sci. Tradit. Chin. Med..

[10] Chen P., Wang Y., Liu Q., Zhang Y., Li X., Li H., Li W. (2020). Phase changes of continuous cropping obstacles in strawberry (*Fragaria × ananassa* Duch.) production. Appl. Soil Ecol..

[11] Clocchiatti A., Hannula S.E., Berg M.V.D., Hundscheid M.P.J., Boer W.D. (2021). Evaluation of phenolic root exudates as stimulants of saptrophic fungi in the rhizosphere. Front. Microbiol..

[12] Tu Y., Shen J., Peng Z., Xu Y., Li Z., Liang J., Wei Q., Zhao H., Huang J. (2023). Biochar-dual oxidant composite particles alleviate the oxidative stress of phenolic acid on tomato seed germination. Antioxidants.

[13] Wu H., Xu J., Wang J., Qin X., Wu L., Li Z., Lin S., Lin W., Zhu Q., Khan M.U. (2017). Insights into the mechanism of proliferation on the special microbes mediated by phenolic acids in the *Radix pseudostellariae* rhizosphere under continuous monoculture regimes. Front. Plant Sci..

[14] Zhou T., Li Q., Huang X., Chen C. (2024). Analysis transcriptome and phytohormone changes associated with the allelopathic effects of ginseng hairy roots induced by different-polarity ginsenoside components. Molecules.

[15] Wu Y., Li X., Yang D., Hu X., Zhang J. (2013). Isolation and identification of the water-soluble components of *Pogostemon cablin*. Chemical Engineering III.

[16] Xu Y., Wu Y., Chen Y., Zhang J., Song X., Zhu G., Hu X. (2015). Autotoxicity in *Pogostemon cablin* and their allelochemicals. Rev. Bras. Farmacogn..

[17] Wu F., Ding Y., Nie Y., Wang X., An Y., Roessner U., Walker R., Du B., Bai J. (2021). Plant metabolomics integrated with transcriptomics and rhizospheric bacterial community indicates the mitigation effects of *Klebsiella oxytoca* P620 on p-hydroxybenzoic acid stress in cucumber. J. Hazard. Mater..

[18] Šoln K., Klemenčič M., Koce J.D. (2022). Plant cell responses to allelopathy: From oxidative stress to programmed cell death. Protoplasma.

[19] Shan Z., Zhou S., Shah A., Arafat Y., Rizvi S.A.H., Shao H. (2023). Plant allelopathy in response to biotic and abiotic factors. Agronomy.

[20] Fadiji A.E., Adeniji A., Lanrewaju A.A., Babalola O.O. (2025). Dynamics of soil microbiome and allelochemical interactions: An overview of current knowledge and prospects. Ann. Microbiol..

[21] Dong W., Wang F., Huang F., Wang Y., Zhou J., Ye X., Li Z., Hou Y., Huang Y., Ma J. (2016). Metabolic Pathway Involved in 6-Chloro-2-Benzoxazolinone Degradation by *Pigmentiphaga* sp. Strain DL-8 and Identification of the Novel Metal-Dependent Hydrolase CbaA. Appl. Environ. Microbiol..

[22] Carrión V.J., Perez-Jaramillo J., Cordovez V., Tracanna V., De Hollander M., Ruiz-Buck D., Mendes L.W., Van Ijcken W.F.J., Gomez-Exposito R., Elsayed S.S. (2019). Pathogen-induced activation of disease-suppressive functions in the endophytic root microbiome. Science.

[23] Revillini D., David A.S., Reyes A.L., Knecht L.D., Vigo C., Allen P., Searcy C.A., Afkhami M.E. (2023). Allelopathy-selected microbiomes mitigate chemical inhibition of plant performance. New Phytol..

[24] Gámiz B., López-Cabeza R., Velarde P., Spokas K.A., Cox L. (2021). Biochar changes the bioavailability and bioefficacy of the allelochemical coumarin in agricultural soils. Pest Manag. Sci..

[25] Zhao X., Elcin E., He L., Vithanage M., Zhang X., Wang J., Wang S., Deng Y., Niazi N.K., Shaheen S.M. (2023). Using biochar for the treatment of continuous cropping obstacle of herbal remedies: A review. Appl. Soil Ecol..

[26] Wu H., Qin X., Wu H., Li F., Wu J., Zheng L., Wang J., Chen J., Zhao Y., Lin S. (2020). Biochar mediates microbial communities and their metabolic characteristics under continuous monoculture. Chemosphere.

[27] Zhao L., Xu W., Guan H., Wang K., Xiang P., Wei F., Yang S., Miao C., Ma L. (2022). Biochar increases *Panax notoginseng*’s survival under continuous cropping by improving soil properties and microbial diversity. Sci. Total Environ..

[28] Ren X., Wang F., Cao F., Guo J., Sun H. (2018). Desorption of atrazine in biochar-amended soils: Effects of root exudates and the aging interactions between biochar and soil. Chemosphere.

[29] Yang B., Pignatello J.J., Qu D., Xing B. (2016). Activation of hydrogen peroxide and solid peroxide reagents by phosphate ion in alkaline solution. Environ. Eng. Sci..

[30] Lu S., Zhang X., Xue Y. (2017). Application of calcium peroxide in water and soil treatment: A review. J. Hazard. Mater..

[31] Wang R., Shi W., Kronzucker H.J., Li Y. (2023). Oxygenation promotes vegetable growth by enhancing P nutrient availability and facilitating a stable soil bacterial community in compacted soil. Soil Tillage Res..

[32] Peng Z., Lin C., Fan K., Ying J., Li H., Qin J., Qiu R. (2024). The use of urea hydrogen peroxide as an alternative N-fertilizer to reduce accumulation of arsenic in rice grains. J. Environ. Manag..

[33] Lu C., Hughes E.W., Giguère P.A. (1941). The crystal structure of the urea-hydrogen peroxide addition compound CO(NH_2_)_2_·H_2_O_2_. J. Am. Chem. Soc..

[34] Cheng J., Zhou C., Xie Y., Wang M., Zhou C., Li X., Du Y., Lu F. (2022). A new method for simultaneous determination of 14 phenolic acids in agricultural soils by multiwavelength HPLC-PDA analysis. RSC Adv..

[35] Xu R., Li A., Tan X., Tang X., He X., Wang L., Kang J., Li S., Liu Y. (2025). Patchouli essential oil extends the lifespan and healthspan of *Caenorhabditis elegans* through JNK-1/DAF-16. Life Sci..

[36] Ni X., Jin C., Liu A., Chen Y., Hu Y. (2021). Physiological and transcriptomic analyses to reveal underlying phenolic acid action in consecutive monoculture problem of *Polygonatum odoratum*. BMC Plant Biol..

[37] Kanjana N., Li Y., Shen Z., Mao J., Zhang L. (2024). Effect of phenolics on soil microbe distribution, plant growth, and gall formation. Sci. Total. Environ..

[38] Yuan M.M., Kakouridis A., Starr E., Nguyen N., Shi S., Pett-Ridge J., Nuccio E., Zhou J., Firestone M., Giovannoni S.J. (2021). Fungal-bacterial cooccurrence patterns differ between arbuscular mycorrhizal fungi and nonmycorrhizal fungi across soil niches. mBio.

[39] Zhang H., Feng H., Zhang C., Zhang X., Jin W., Liang Z. (2021). Identification of phenolic acids in rhizosphere soil of continuous cropping of *Salvia miltiorrhiza* Bge. Allelopath. J..

[40] Zhang B., Weston L.A., Li M., Zhu X., Weston P.A., Feng F., Zhang B., Zhang L., Gu L., Zhang Z. (2020). *Rehmannia glutinosa* replant issues: Root exudate-rhizobiome interactions clearly influence replant success. Front. Microbiol..

[41] Liao J., Xia P. (2024). Continuous cropping obstacles of medicinal plants: Focus on the plant-soil-microbe interaction system in the rhizosphere. Sci. Hortic..

[42] Zhao L., Guan H., Wang R., Wang H., Li Z., Li W., Xiang P., Xu W. (2021). Effects of tobacco stem-derived biochar on soil properties and bacterial community structure under continuous cropping of *Bletilla striata*. J. Soil Sci. Plant Nutr..

[43] Lin C., Liu Y., Chang P., Hsieh Y., Tzou Y. (2023). Inhibition of continuous cropping obstacle of celery by chemically modified biochar: An efficient approach to decrease bioavailability of phenolic allelochemicals. J. Environ. Manag..

[44] Zhang C., Zhang Q., Luo M., Wang Q., Wu X. (2023). *Bacillus cereus* WL08 immobilized on tobacco stem charcoal eliminates butylated hydroxytoluene in soils and alleviates the continuous cropping obstacle of *Pinellia ternata*. J. Hazard. Mater..

[45] Gan T., Yuan Z., Gustave W., Luan T., He L., Jia Z., Zhao X., Wang S., Deng Y., Zhang X. (2025). Challenges of continuous cropping in *Rehmannia glutinosa*: Mechanisms and mitigation measures. Soil Environ. Health.

[46] Ravi S., Young T., Macinnis-Ng C., Nyugen T.V., Duxbury M., Alfaro A.C., Leuzinger S. (2020). Untargeted metabolomics in halophytes: The role of different metabolites in New Zealand mangroves under multi-factorial abiotic stress conditions. Environ. Exp. Bot..

[47] Xu L., Ma L., Wei R., Ma Y., Ma T., Dang J., Chen Z., Li S., Ma S., Chen G. (2024). Effect of continuous cropping on growth and lobetyolin synthesis of the medicinal plant *Codonopsis pilosula* (Franch.) Nannf. based on the integrated analysis of plant-metabolite-soil factors. J. Agric. Food Chem..

[48] Zhou Y., Liu Y., Jiang C., El-Desouki Z., Riaz M., Wang C., Zhang X., Ding J., Chen Z., Liu H. (2025). Effects of exogenous application of phenolic acid on soil nutrient availability, enzyme activities, and microbial communities. Agriculture.

[49] Xie A., Li X., Zhang D., Yang X., Shi Y., Dong L., Lei F., Lv M., Sun L., Sun X. (2025). Changes in herbaceous peony growth and rhizosphere soil after benzoic and gallic acids application. Sci. Hortic..

[50] Irfan M., Hussain Q., Khan K.S., Akmal M., Ijaz S.S., Hayat R., Khalid A., Azeem M., Rashid M. (2019). Response of soil microbial biomass and enzymatic activity to biochar amendment in the organic carbon deficient arid soil: A 2-year field study. Arab. J. Geosci..

[51] Lan P., Chen Q., Lu M., Steinberg C.E.W., Wu M. (2023). Biochar reduces generation and release of benzoic acid from soybean root. J. Soil Sci. Plant Nutr..

[52] Jin F., Piao J., Miao S., Che W., Li X., Li X.B., Shiraiwa T., Tanaka T., Taniyoshi K., Hua S. (2024). Long-term effects of biochar one-off application on soil physicochemical properties, salt concentration, nutrient availability, enzyme activity, and rice yield of highly saline-alkali paddy soils: Based on a 6-year field experiment. Biochar.

[53] Wang J., Zhang X., Zhou X., Waigi M.G., Gudda F.O., Zhang C., Ling W. (2021). Promoted oxidation of polycyclic aromatic hydrocarbons in soils by dual persulfate/calcium peroxide system. Sci. Total Environ..

[54] Kamali M., Sweygers N., Al-Salem S., Appels L., Aminabhavi T.M., Dewil R. (2022). Biochar for soil applications-sustainability aspects, challenges and future prospects. Chem. Eng. J..

[55] Han J., Li Y., Li H., Yang H., Luo S., Man H., Shi G. (2025). Phenolic acids alleviated consecutive replant problems in lily by regulating its allelopathy on rhizosphere microorganism under chemical fertiliser reduction with microbial agents in conjunction with organic fertiliser application. Appl. Soil Ecol..

[56] Wang C., Kuzyakov Y. (2024). Mechanisms and implications of bacterial-fungal competition for soil resources. ISME J..

[57] Yao J., Wu C., Fan L., Kang M., Liu Z., Huang Y., Xu X., Yao Y. (2023). Effects of the Long-term continuous cropping of Yongfeng yam on the bacterial community and function in the rhizospheric soil. Microorganisms.

[58] Fan Q., Fan X., Fu P., Li Y., Zhao Y., Hua D. (2022). Anaerobic digestion of wood vinegar wastewater using domesticated sludge: Focusing on the relationship between organic degradation and microbial communities (archaea, bacteria, and fungi). Bioresour. Technol..

[59] Hong L., Yao Y., Lei C., Hong C., Zhu W., Zhu F., Wang W., Lu T., Qi X. (2023). Declined symptoms in *Myrica rubra*: The influence of soil acidification and rhizosphere microbial communities. Sci. Hortic..

[60] Xu W., Li H., Ma Q., Dong Q., Gao J., Zhang F., Xie H. (2025). Mitigating continuous cropping challenges in alkaline soils: The role of biochar in enhancing soil health, microbial dynamics, and pepper productivity. Ind. Crops Prod..

[61] Xiao R., Lei H., Zhang Y., Xiao Z., Yang G., Pan H., Hou Y., Yu J., Sun K., Dong Y. (2023). The influence of aerated irrigation on the evolution of dissolved organic matter based on three-dimensional fluorescence spectrum. Agronomy.

[62] Chen J., Liu Z., Liu Y., Ji X., Li X., Wei Y., Zi F., Tan Y. (2024). Differences in autotoxic substances and microbial community in the root space of *Panax notoginseng* coinducing the occurrence of root rot. Appl. Environ. Microbiol..

[63] Wang G., Yin C., Pan F., Wang X., Xiang L., Wang Y., Wang J., Tian C., Chen J., Mao Z. (2018). Analysis of the fungal community in apple replanted soil around Bohai Gulf. Hortic. Plant J..

[64] Sharma-Poudyal D., Schlatter D., Yin C., Hulbert S., Paulitz T. (2017). Long-term no-till: A major driver of fungal communities in dryland wheat cropping systems. PLoS ONE.

[65] Ge S., Gao J., Chang D., He T., Cai H., Wang M., Li C., Luo Z., E Y., Meng J. (2023). Biochar contributes to resistance against root rot disease by stimulating soil polyphenol oxidase. Biochar.

[66] Stepanov A.A., Shulaev N.A., Vasilchenko A.S. (2025). The Ecological Strategy Determines the Response of Fungi to Stress: A Study of the 2,4-diacetylphloroglucinol Activity Against Aspergillus and Fusarium Species. J. Basic Microbiol..

[67] Xu D., Yu X., Zhang Y., Liu Y., Chen C., Li L., Fan S., Lu X., Zhang X. (2025). Effect of compost as a soil amendment on the structure and function of fungal diversity in saline-alkali soil. Curr. Res. Microb. Sci..

