# Supplementary material for: Biochar–Urea Peroxide Composite Particles Alleviate Phenolic Acid Stress in Pogostemon cablin Through Soil Microenvironment Modification
Source: Microorganisms. 2025 Dec 5;13(12):2772. doi: 10.3390/microorganisms13122772 (PMC12735811; doi:10.3390/microorganisms13122772)
Supplement: Supplementary file 1 [file microorganisms-13-02772-s001.zip › microorganisms-3957899-supplementary.pdf]

Supplementary data

Biochar–Urea Peroxide Composite particles alleviate Phenolic Acid Stress in *Pogostemon cablin* through Soil Microenvironment Modification

Yuting Tu [1,2,3,*,†], Baozhu Chen [1,4,†], Qiufang Wei [1,5], Yanggui Xu [1,2,3], Yiping Peng [1,2,3], Zhuxian Li [1,2,3], Jianyi Liang [1,2,3], Lifang Zhuo [1,5], Wenliang Zhong [1,2,3] and Jichuan Huang [1,2,3,*]

[1] Institute of Agricultural Resources and Environment, Guangdong Academy of Agricultural Sciences, Guangzhou 510640, China; chenbaozhu@stu.gdou.edu.cn (B.C.); qfwei@stu.scau.edu.cn (Q.W.); xuyanggui@gdaas.cn (Y.X.); pengyiping@gdaas.cn (Y.P.); lizhuxian@gdaas.cn (Z.L.); liangjianyi@gdaas.cn (J.L.); folklore1998@stu.scau.edu.cn (L.Z.); zhongwenliang@gdaas.cn (W.Z.)
[2] Key Laboratory of Plant Nutrition and Fertilizer in South Region, Ministry of Agriculture and Rural Affairs of the People's Republic of China, Guangzhou 510640, China
[3] Guangdong Key Laboratory of Nutrient Cycling and Farmland Conservation, Guangzhou 510640, China
[4] School of Chemistry and Environment, Guangdong Ocean University, Zhanjiang 524088, China
[5] The College of Natural Resources and Environment, South China Agricultural University, Guangzhou 510642, China
* Correspondence: tuyuting@gdaas.cn (Y.T.); huangjichuan@gdaas.cn (J.H.); Tel.: +86-20-38469763 (J.H.)
† These authors contributed equally to this work.

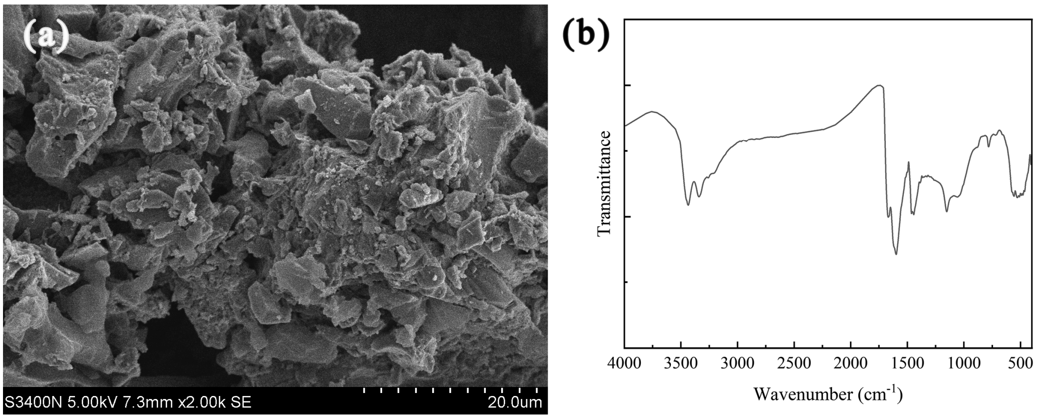

Figure S1. SEM micrograph and FTIR spectra of BC-UP composite particles.

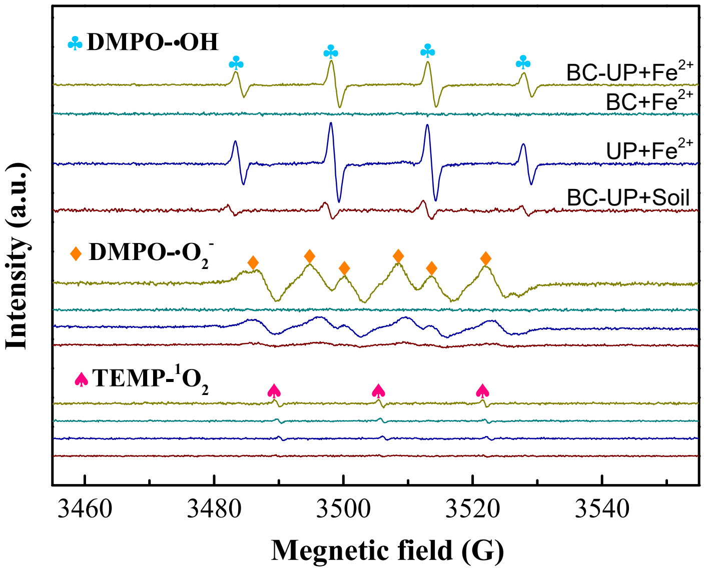

Figure S2. Comparison of electron paramagnetic resonance (EPR) spectra of ROS trapped by DMPO and TEMP in different treatment systems. Reaction conditions: 100 mg/L p-CA, 25 °C, 10 min. Treatment systems: BC-UP+Fe^{2+} (10 g/L BC-UP, 0.2 mmol/L Fe^{2+}), BC+Fe^{2+} (8 g/L BC, 0.2 mmol/L Fe^{2+}), UP+Fe^{2+} (2 g/L UP, 0.2 mmol/L Fe^{2+}), BC-UP+Soil (10 g/L BC-UP, 2 g/L soil).

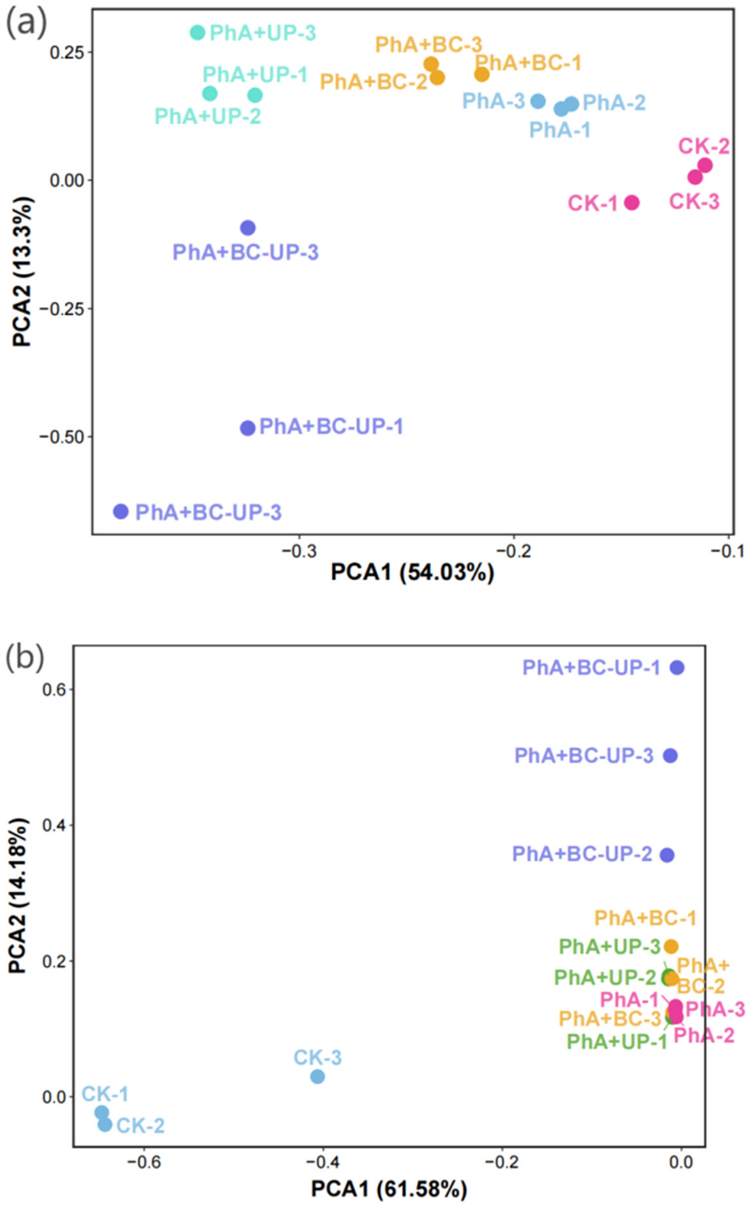

Figure S3. PCA of bacterial (**a**) and fungal (**b**) communities across different exogenous additive treatments.

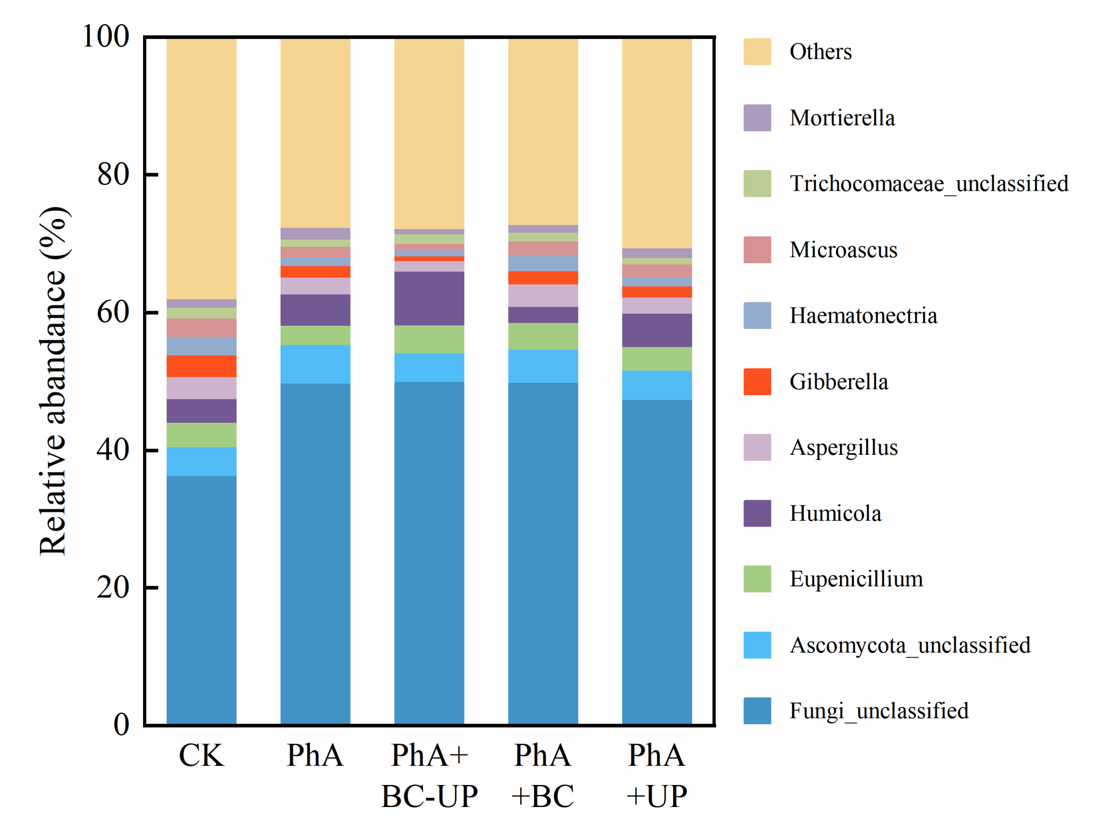

Figure S4. Fungal community structure at the genus level in patchouli rhizosphere soil across treatments.

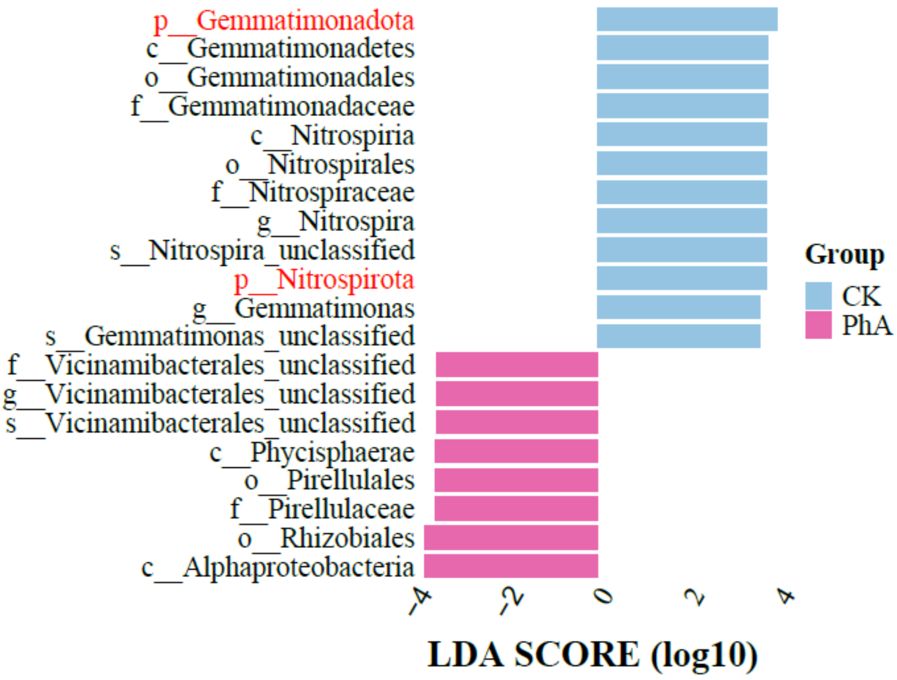

Figure S5. Bar plots of LDA scores from LEfSe analysis identifying differentially bacterial taxa in rhizosphere soil between CK and PhA treatments.

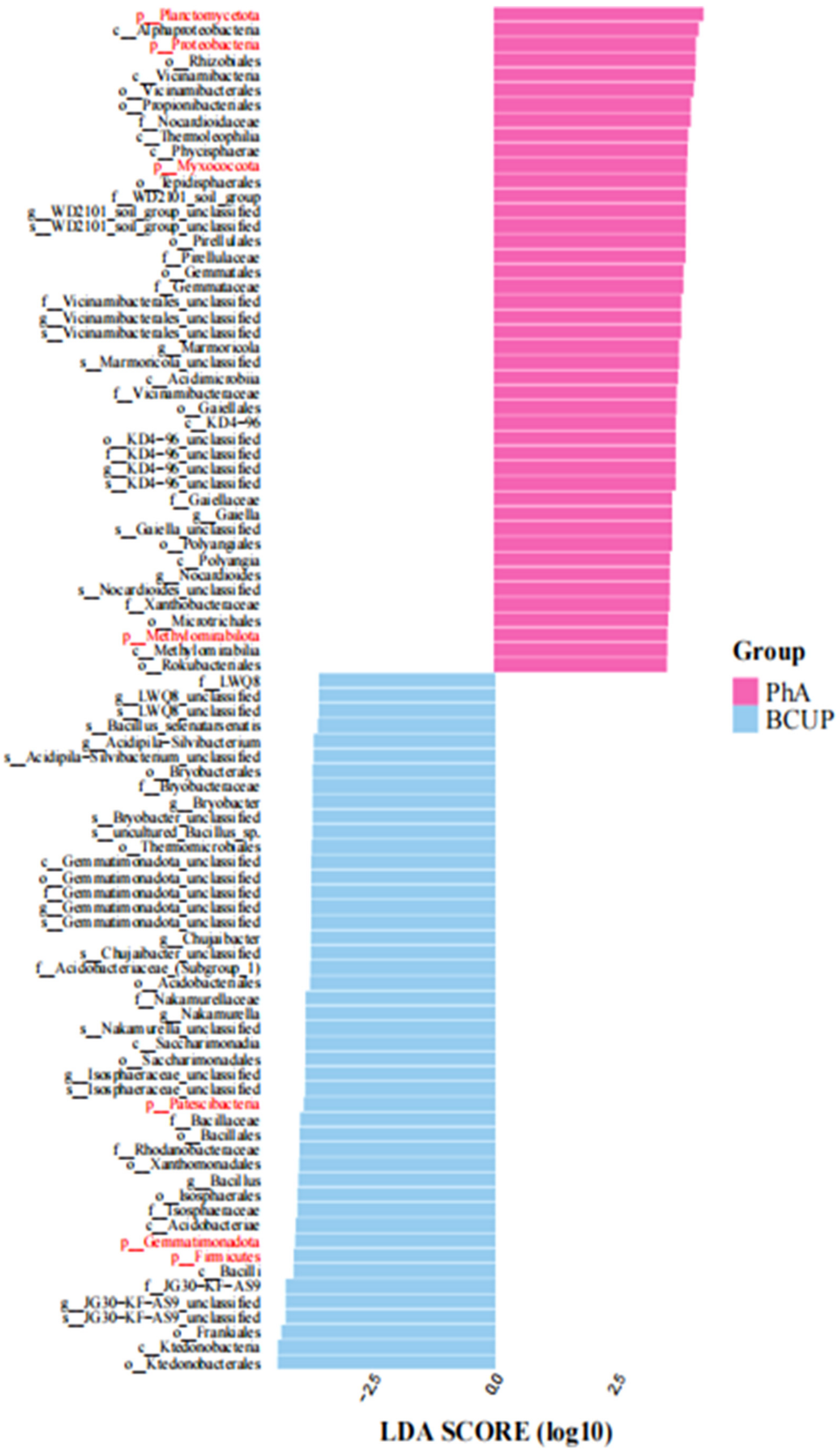

Figure S6. Bar plots of LDA scores from LEfSe analysis identifying differentially bacterial taxa in rhizosphere soil between PhA and composite additive treatments.

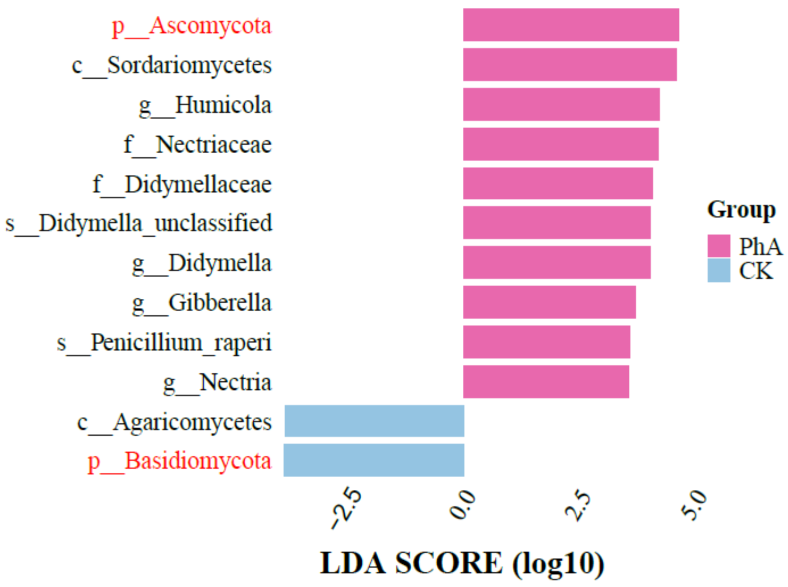

Figure S7. Bar plots of LDA scores from LEfSe analysis identifying differentially fungal taxa in rhizosphere soil between CK and PhA treatments.

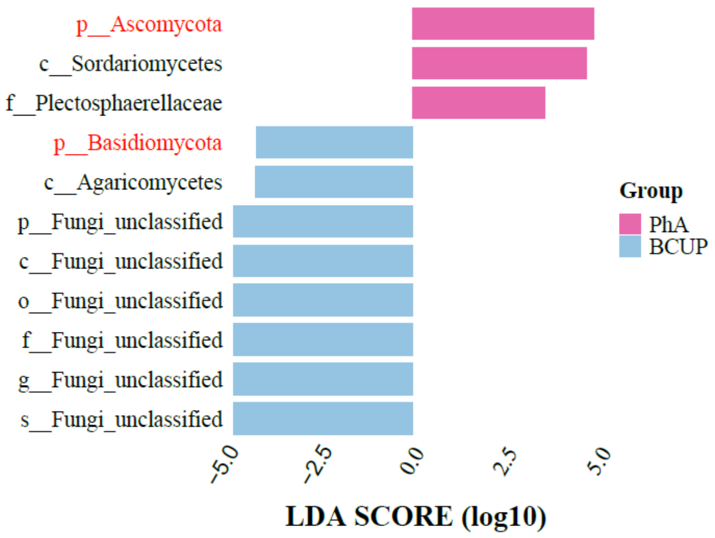

Figure S8. Bar plots of LDA scores from LEfSe analysis identifying differentially fungal taxa in rhizosphere soil between PhA and composite additive treatments.

Table S1. Growth parameters of *Pogostemon cablin* under different addition amounts of BC-UP composite particles.

Treatment	Shoot Fresh Weight (g)	Root Fresh Weight (g)	Plant Height (cm)	Root Length (cm)
CK	24.66±1.43 a	1.64±0.09 b	31.04±0.49 a	15.81±0.61 b
PhA	18.46±0.07 bc	1.43±0.1 c	22.23±1.56 d	13.79±0.84 c
PhA+2.5 BC-UP	18.95±0.81 b	1.38±0.09 c	22.33±0.58 d	15.90±0.56 b
PhA+5.0 BC-UP	23.77±0.25 a	2.40±0.02 a	26.99±1.4 b	18.18±0.22 a
PhA+10.0 BC-UP	17.35±0.05 c	2.29±0.08 a	24.20±0.78 c	17.47±0.27 a
PhA+15.0 BC-UP	5.43±0.28 d	0.97±0.01 d	17.20±0.65 e	14.17±0.29 c

Data are presented as mean ± SD (n=3). Different lowercase letters within a column indicate significant differences between treatments according to Duncan's multiple range test ($p < 0.05$).
